# Novel druggable space in human KRAS G13D discovered using structural bioinformatics and a P-loop targeting monoclonal antibody

**DOI:** 10.1038/s41598-024-70217-9

**Published:** 2024-08-23

**Authors:** Oscar Jungholm, Carolina Trkulja, Martin Moche, Sreesha P. Srinivasa, Maria-Nefeli Christakopoulou, Max Davidson, Anna Reymer, Kent Jardemark, Rafaela Lenza Fogaça, Anaswara Ashok, Gavin Jeffries, Henry Ampah-Korsah, Emilia Strandback, Juni Andréll, Tomas Nyman, Ghada Nouairia, Owe Orwar

**Affiliations:** 1 Department of Physiology and Pharmacology, Karolinska Institutet, 171 77 Stockholm, Sweden; 2 Oblique Therapeutics AB, 41346 Gothenburg, Sweden; 3 Department of Chemistry and Molecular Biology, University of Gothenburg, 405 30 Gothenburg, Sweden; 4 Fluicell AB, Flöjelbergsgatan 8C, 431 37 Mölndal, Sweden; 5 Protein Science Facility, Karolinska Institutet, 171 77 Stockholm, Sweden; 6 Department of Medicine Huddinge, Karolinska Institutet, 171 77 Stockholm, Sweden; 7 Present Address: Fluicell AB, Flöjelbergsgatan 8C, 431 37 Mölndal, Sweden; 8 Present Address: Department of Physiology and Pharmacology, Karolinska Institutet, 171 77 Stockholm, Sweden; 9 Present Address: Manipal Center for Biotherapeutics Research, Manipal Academy of Higher Education, Manipal, India

**Keywords:** Molecular modelling, Oncogenes, X-ray crystallography, Cell delivery

## Abstract

KRAS belongs to a family of small GTPases that act as binary switches upstream of several signalling cascades, controlling proliferation and survival of cells. Mutations in KRAS drive oncogenesis, especially in pancreatic, lung, and colorectal cancers (CRC). Although historic attempts at targeting mutant KRAS with small molecule inhibitors have proven challenging, there are recent successes with the G12C, and G12D mutations. However, clinically important RAS mutations such as G12V, G13D, Q61L, and A146T, remain elusive drug targets, and insights to their structural landscape is of critical importance to develop novel, and effective therapeutic concepts. We present a fully open, P-loop exposing conformer of KRAS G13D by X-ray crystallography at 1.4–2.4 Å resolution in Mg^2+^-free phosphate and malonate buffers. The G13D conformer has the switch-I region displaced in an upright position leaving the catalytic core fully exposed. To prove that this state is druggable, we developed a P-loop-targeting monoclonal antibody (mAb). The mAb displayed high-affinity binding to G13D and was shown using high resolution fluorescence microscopy to be spontaneously taken up by G13D-mutated HCT 116 cells (human CRC derived) by macropinocytosis. The mAb inhibited KRAS signalling in phosphoproteomic and genomic studies. Taken together, the data propose novel druggable space of G13D that is reachable in the cellular context. It is our hope that these findings will stimulate attempts to drug this fully open state G13D conformer using mAbs or other modalities.

## Introduction

The RAS family of small GTPases is the most frequently mutated oncogene in all human cancer^1^. Activating mutations are highly lethal and most frequently found in KRAS (85%), less commonly in NRAS (12%) and least in HRAS (3%)^1–3^. KRAS exists in two splice variants 4A, and 4B, that are often co-expressed in various malignancies, and 4B is generally considered to be more oncogenic^4–6^. Small molecule inhibitors, such as Sotorasib (AMG 510), have recently demonstrated the clinical utility of targeting KRAS for patient benefit^7–9^. These drugs are selective for the KRAS G12C mutation where glycine has been substituted with a cysteine at the twelfth position and exploit the reactivity of this cysteine for covalent binding^9^. There has also been a recent breakthrough showing potent and selective in vitro and in vivo activity against the G12D mutation by a small molecule approach^10^. Despite this success, targeting clinically important RAS mutations such as, G12V, G13D, Q61L, and A146T, have been problematic due to the lack of a reactive amino acid residue or hydrophobic pockets in the protein^11^. Upon activation by upstream growth factor receptors, RAS undergoes exchange of guanosine 5ʹ diphosphate (GDP) for guanosine triphosphate (GTP) with the help of a guanine nucleotide exchange factor (GEF) such as son of sevenless 1 (SOS1)^12,13^. In the GTP-bound state, RAS can interact, possibly as a dimer^14–17^ with several survival-promoting effectors, e.g., PI3K, Raf etc., thereby activating downstream signalling cascades resulting in regulation of these biological processes. With the help of GTPase-activating protein (GAP), GTP is hydrolysed to GDP thereby desensitizing the signalling cascades. Activating mutations in RAS lock the protein in a GTP-bound ‘on’ state, driving uncontrolled growth and survival programs thus initiating fundamental hallmarks of oncogenesis^2,3^. Nucleotide exchange involves a transient state where the switch-I is opened, a process in large part driven by SOS1^12,13^. HRAS G13D^18^, KRAS G13D^19^, and KRAS A146T^20^ mutant proteins stand out with several-fold higher nucleotide exchange rates compared to wild type or other common RAS mutants indicating a higher capacity for GEF-independent autoactivation and a lower energy barrier for switch-I opening. For example, Hunter et al., found G13D to have a constitutive, as well as SOS1-assisted GDP exchange rate about 10–15 times faster, compared to WT, G12A, G12C, G12S, G12D, G12V, Q61L, and Q61H variants^19^. Interestingly, G13D and A146T mutations seem to be associated with higher tumour burden and lower survival in CRC patients^21–23^. Kim et al., recently demonstrated effective inhibition of several RAS mutants, including G13D, and A146V/T, as well as WT KRAS by a small molecule approach, resulting in nucleotide exchange interference^24^. Khan et al., also exploited differential nucleotide exchange rates in KRAS mutants using a monobody approach^25^. By targeting the nucleotide unbound apo state, the authors successfully inhibited mutated KRAS G13D, A146T, G12D and Q61L that was rationalized in part by their high intrinsic nucleotide exchange rates.

Out of the near 470 KRAS structures in the Protein Data Bank (PDB), ten different Mg^2+^-free KRAS structures with fully open switch-I have been deposited. For example, A146T (PDB entry: 6BOF) and WT KRAS (PBD entry: 6M9W) where switch-I is extended far away from the catalytic core leaving the P-loop area fully exposed, have been described historically using X-ray crystallography^20,26–28^. We will refer to these conformers as “fully open” with the P-loop exposed in contrast to known “semi-open”, Mg^2+^-bound mutants such as 6ASA^26^ (D33E), 6ASE^26^ (A59G), and 8FMK^27^ (G12D) where the P-loop area is less exposed, and “closed” structures such as 4TQA^19^ (G13D) which are Mg^2+^-bound and the P-loop area is hidden. This terminology resonates with that used by Dharmaiah et al.^28^*.*

To understand what structural transformations that are plausible for G13D in vivo, the first objective of the present study was to investigate if Mg^2+^-free G13D can exist as a fully open state, P-loop exposing conformer. We used X-ray crystallography to identify this “fully open” G13D conformer, exposing the P-loop region. Even if X-ray crystallographic data is obtained under far from in vivo conditions, the data suggests novel druggable space within the catalytic core in the P-loop region. The second objective was to demonstrate that this conformer can bind a P-loop targeting mAb, and for this reason a novel mAb was developed using a proteins-in-motion proteomics technology^29^. Finally, we demonstrate that uptake of the mAb into cancer cells results in inhibition of KRAS signalling, in vitro, suggesting that an antibody-based approach to target the fully open state of G13D is a compelling and exciting possibility for future therapeutics.

### A fully open switch-I conformational state of KRAS G13D discovered by X-ray crystallography

**Figure 1:**
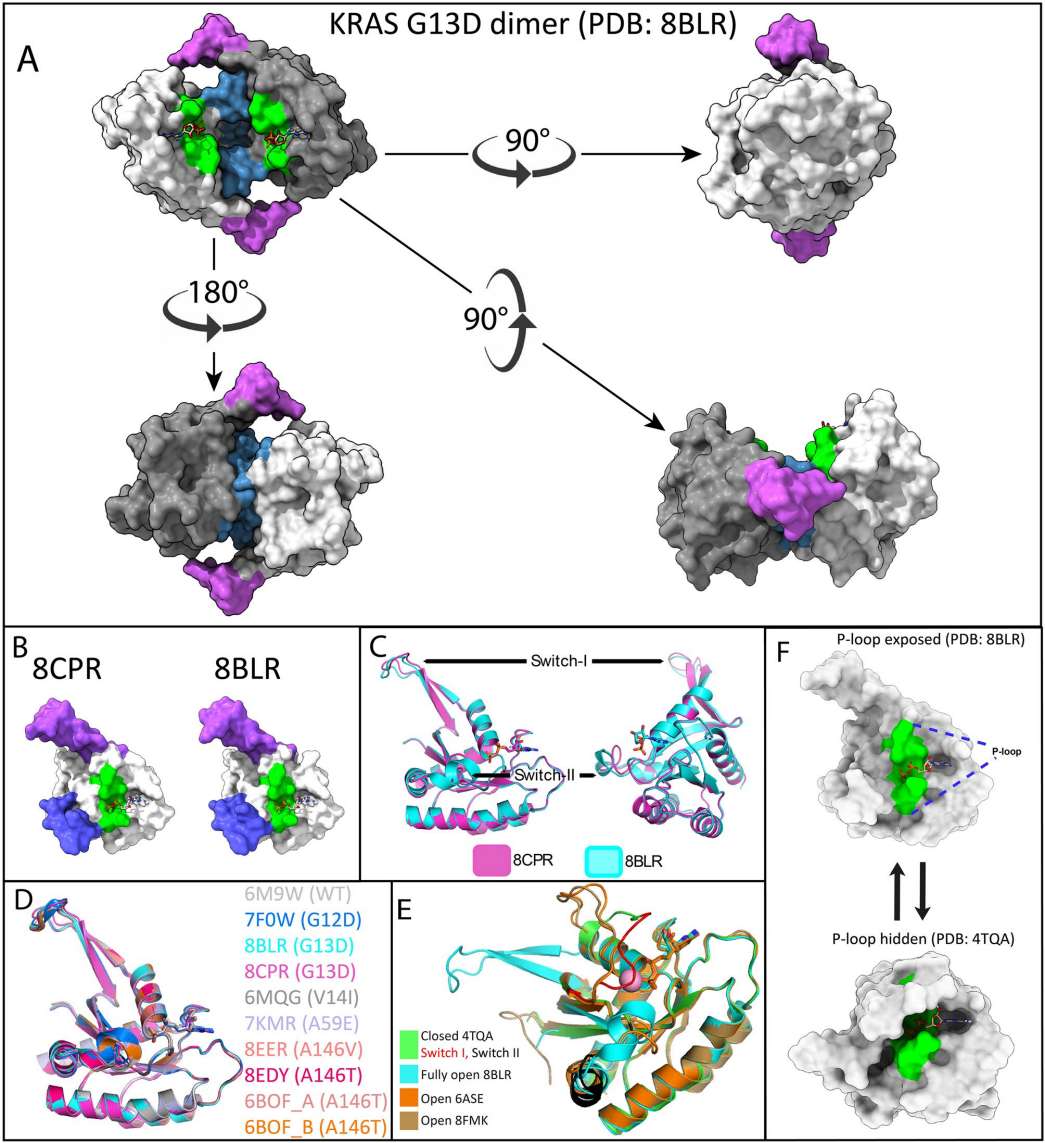
X-ray crystallography of open KRAS variants. (**A**) Solvent-excluded surface representation of the hypothetical KRAS dimer for 8BLR visualized in four poses. The dimeric state of KRAS was assigned by the PISA server. switch-I surface is coloured in magenta, switch-II in blue, and the P-loop in green. GDP in ball-and-stick representation. (**B**) Solvent-excluded surface representation of fully open KRAS monomer for 8CRP (left) and 8BLR (right). (**C**) Superposition of KRAS monomers, 8CPR in magenta and 8BLR in cyan, in a cartoon secondary structure representation, showing the close similarity between the two models except in the switch-I and switch-II regions, where small differences are seen. (**D**) Multiple structural alignment of all ten known fully open monomeric KRAS chains in the protein data bank (PDB) using PDBeFold. Every fully open KRAS structure in PDB is between 167 and 171 amino acids in length and can be aligned with an overall RMSD (root mean square deviation) of 0.408, and an overall Q-score of 0.947 for 165 matching residues. None of the fully open KRAS structures have Mg^2+^ in the active site (see also Supplementary Table S2). (**E**) KRAS, switch-I has been observed in three major conformations referred to as closed, semi-open, and fully open conformation. Here the closed form is represented by 4TQA, a G13D mutant coloured in green except for switch-I (here 30–38) coloured in red and switch-II (here 60–76) coloured in black. The semi-open conformation is represented by 8FMK, a G12D mutant coloured in sand, and 6ASE, an A59G mutant coloured in orange. The fully open conformation is represented by our G13D mutant 8BLR structure coloured in cyan. Please note that the Mg^2+^ in the active site are drawn as pink spheres.. (**F**) X-ray crystallography structures of KRAS G13D showing switch-I in fully open conformation (PDB: 8BLR) and switch-I in closed conformation (PDB: 4TQA) the P-loop is highlighted in green.

In the present study, we used the KRAS4A splice variant, which is denoted as KRAS G13D throughout the article. KRAS4A is distinguished from KRAS4B by having distinct membrane-targeting sequences, but they share a high degree of sequence similarity in the catalytic P-loop region^5^. KRAS4A is oncogenic just like KRAS4B and is expressed in HCT 116 cells (ca 20% of total KRAS) and, more importantly, KRAS4A is highly expressed, up to 50% of total KRAS in 17 human colorectal tumors^5^. For X-ray crystallographic- and ELISA studies, we produced G13D KRAS4A (2–169) lacking the initiator methionine (iMet) in magnesium free buffer, with the intention to create a Mg^2+^-free fully open state G13D conformer. The iMet deletion approach was inspired by the work of Dharmaiah et al.^28^ who successfully crystalized a switch-I fully open conformer of wtKRAS in Mg^2+^-free media. We determined the x-ray crystallographic 3D structure of KRAS G13D in sodium-malonate pH 7 buffer (PDB entry: 8BLR), and in sodium–potassium phosphate pH 8.7 buffer (PDB entry: 8CPR), respectively. Both 8BLR and 8CPR crystallized as dimers with switch-I in fully open conformation. The malonate crystals diffracted to 1.4 Å resolution in all directions (isotropic) and the phosphate crystals to 2.0/2.4 Å in best/worst direction (anisotropic). Both 8BLR and 8CPR were confirmed to represent a G13D mutant of KRAS4A (2–169) bound to GDP with the switch-I in fully open conformation. Data collection and refinement statistics for KRAS4A-G13D (8BLR & 8CPR) can be found in Supplementary Table S1. The 8BLR dimer is shown in four different poses in Fig. 1A, where one monomer is white-coloured and the other monomer grey-coloured while both monomers are having the P-loop coloured in green, switch-I (aa 30–40) coloured in magenta, and switch-II (aa 58–72) coloured in blue (see also Supplementary Video S1). The respective monomeric forms of 8CPR and 8BLR are shown in the left and right panels, respectively in Fig. 1B exposing the P-loop areas (green) and having their switch-I regions (magenta) and switch-II regions (blue) opened (see also Supplementary Videos S2 and S3 respectively). Two different poses, showing alignments of 8BLR and 8CPR can be seen in Fig. 1C (see also Supplementary Video S4), comparing 8BLR and 8CPR backbones. Most noteworthy is the apparent displacement of the switch-II region that can be considered a dynamic hotspot region when looking at electron densities. We observed several switch-II side-chains such as Asp57, Gln61, and Glu63 in multiple conformations in the higher-resolution 8BLR structure. In Fig. 1D, we compare 8CPR and 8BLR to the other eight fully open and Mg^2+^-free models previously deposited in the protein data bank. These are represented by A146T (PDB entry: 6BOF^20^, 8EDY to be published), A146V (PDB entry: 8EER to be published), G12D (PDB entry: 7F0W to be published), V14I (PDB entry: 6MQG^30^), A59E (PDB entry: 7KMR^31^) and WT KRAS (PBD entry: 6M9W^28^). Counting 8BLR and 8CPR, there are now ten PDB entries of fully open form KRAS, and one entry (6BOF) deviates from other fully open structures by containing two molecules in the asymmetric unit (6BOF_A & 6BOF_B in Fig. 1D). The ten fully open conformers are observed in six different KRAS mutations with representatives from both splice variants. The length of the ten fully open KRAS chains are all between 167 and 171 amino acids and can be aligned with an overall root mean square deviation of 0.4082 for 166 aligned residues using PDBeFold^2^, further indicating that all fully open KRAS structures are very similar to each other. Our first impression of 8CPR hinted towards a unique conformation for a few residues belonging to switch II (60–76), however, b-factors are high in the area and electron density indicates flexibility in this part of 8CPR. In Fig. 1E we compare the switch-I closed 4TQA^19^ (G13D), to semi-open, 6ASE^26^ (A59G), and 8FMK^27^ (G12D), and to the fully open 8BLR (G13D) conformer. The closed and semi-open forms retain the Mg^2+^ ion in the active site while all fully open structures including 8BLR and 8CPR observed herein are all presented without Mg^2+^ in the active site. We believe that G13D has a high probability for having a fully open form by electrostatically repelling the negatively charged switch-I loop that closes over the nucleotide and codon 13 aspartate. In this open-state conformation, the P-loop-located region is exposed and should be accessible for binding mAbs, peptides, or small molecules. In the cellular context, we hypothesize that G13D is cycling between one or possibly several switch-I fully open states, and a switch-I closed state (see e.g. 4TQA) as schematically displayed in Fig. 1F. Additionally, there might exist a third Mg^2+^-bound semi-open state of G13D similar to 6ASA^26^ (D33E), 6ASE^26^ (A59G), and 8FMK^27^ (G12D) (exemplified in Fig. 1E) as well as the G13D dimer, and apo forms. Whereas the existence of all these conformations in vivo remains to be proven, the palette of plausible conformers is fascinating. For certain, G13D is required to be in the fully open state to allow for nucleotide exchange, and as already mentioned above it has been observed that nucleotide exchange rates in G13D-mutated HRAS, and KRAS are several fold higher compared to WT KRAS and other common mutations^18,19^. This presents an opportunity to develop G13D P-loop-targeting inhibitors.

### A monoclonal antibody binds a switch-I open conformational state of KRAS G13D

**Figure 2:**
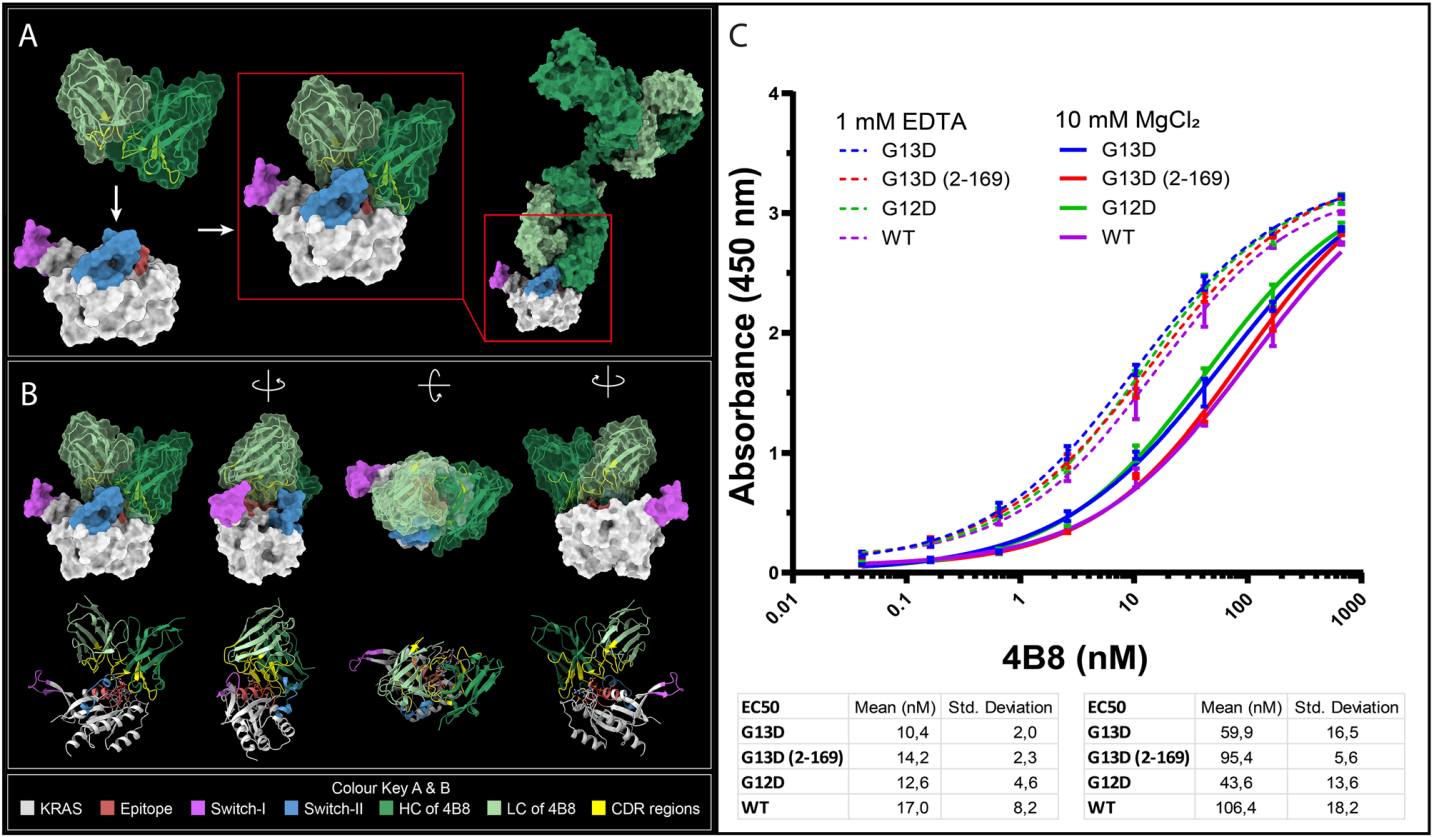
Molecular docking simulations of 4B8 to KRAS G13D with switch-I in fully open conformation, and ELISA binding assays of KRAS proteins in Mg^2+^-containing, and Mg^2+^-free buffer, respectively. (**A**) 4B8 binding to KRAS G13D (PDB: 8BLR) with switch-I in fully open conformation (left) as shown by a docking pose displaying the Fv region of 4B8 interacting with the region 10–21 of human KRAS G13D (middle). Docking pose displaying the full length 4B8 antibody interacting with the region 10–21 of human KRAS G13D (right). (**B**) Docking pose displaying the Fv region of 4B8 antibody interacting with the region 10–21 of human KRAS G13D protein, in different orientations as depicted by the arrows, shown with surface models (top) and ribbon structures (bottom). Colour key for A and B: Crystal structure of KRAS G13D 8BLR with the switch-I in fully open conformation (grey). Epitope region (aa 10–21, red). switch-I (aa30-40, purple), switch-II (aa 58–72, blue). 4B8 heavy chain (dark green). 4B8 light chain (light green). CDR regions (yellow). (**C**) Binding of 4B8 to G13D, G13D 2–169, G12D and wt KRAS with and without Mg^2+^. Measurement of 4B8 binding to G13D, G13D 2–169, G12D and wt KRAS protein by ELISA in either 20 mM Tris, 50 mM NaCl, 1 mM EDTA (dotted lines) or 20 mM Tris, 50 mM NaCl, 10 mM MgCl_2_ (solid lines). Data is presented as mean ± SEM, *n* = 3. One-way ANOVA with Turkey’s multiple comparison test revealed the following significant differences: There was a statistically significant difference (*p* < 0.05) in binding of 4B8 (EC50) between the 1 mM EDTA and the 10 mM MgCl_2_ groups for each of the proteins except G12D. Within the EDTA-treated group, there was no statistically significant difference in binding of 4B8 to any of the four proteins. In the 10 mM MgCl_2_ group, there was a statistically significant difference (*p* < 0.05) in binding of 4B8 between WT and G13D, WT and G12D, G13D and G13D 2–169, and G12D and G13D 2–169.

We developed a small library of P-loop targeting antibodies using peptide antigens corresponding to P-loop residues 10–21 in the G13D KRAS mutation. The peptide antigens were identified using a mAb proteomics discovery technology that we developed previously^29^. In this approach, proteases serve as surrogate antibodies, and if a protease bind to a certain epitope, in this case in P-loop-exposed KRAS, there is a high probability that an antibody will bind to the very same epitope under live conditions. Antibodies were generated by using hybridoma technology whereby mice were immunised withthese P-loop derived peptide antigens. Recombinant antibodies were subsequently produced in Chinese hamster ovary (CHO) cells. Screening of antibody binding against peptide-antigens was performed using ELISA, and a monoclonal antibody, 4B8 was found to be of particular interest as it binds G13D, G12D, as well as WT peptide with EC50 values in the low nM range (Supplementary Fig. S1). Molecular docking simulations were performed using Piper (BioLuminate, Schrödinger Suites of Programs) to confirm accessibility to the P-loop by an IgG mAb to KRAS in the fully open state. Here, the higher resolution structure 8BLR was used, with and without GDP present. The percentage of antibody-docked poses that showed interaction was determined to be 44% and 69%, with and without GDP present, respectively. In comparison, docking simulations against fully open wt KRAS (6M9W) showed interactions for 14% and 42%, respectively, and docking simulations against fully open G12D KRAS (7F0W) showed interactions for 17% and 25%, respectively. This result indicates a stronger binding interaction for the monoclonal antibody to fully open-state G13D compared to wt KRAS and G12D KRAS. Chi-square (χ^2^) tests were performed for the docking simulations comparing binding to KRAS with and without GDP present, showing that there is a statistically significant difference in antibody binding to all three KRAS structures with and without GDP present (χ^2^ (8BLR) = 11.9, χ^2^ (6M9W) = 40.8, χ^2^ (7F0W) = 51.4). Further, Chi-square (χ^2^) tests were performed for the docking simulations comparing binding to G13D, G12D and wt KRAS, independently of the presence of a co-factor. The calculated total chi-square value (93.06) was much greater than the critical value (5.99), showing that there is a statistically significant difference in the binding of the antibody to the three different KRAS structures (see Supplementary Statistics). Docking poses showing interaction between the monoclonal antibody and KRAS G13D structure 8BLR, with GDP present, are displayed in Fig. 2A,B, Supplementary Fig. S2 and Supplementary Video S5 showing the Fragment variable (Fv) region of the monoclonal antibody (green) with the CDRs (yellow) aligning and interacting with the epitope (red). Potentially, by binding to the epitope overlapping the P-loop, 4B8 could prevent switch-I from closing. This would lock KRAS in an inactive transition state by preventing the formation of the effector-binding region, i.e., the interaction interface for downstream effectors proposed to be formed when switch-I and switch-II converge, is blocked^32–34^. Next, we performed ELISA assays using Mg^2+^-containing, and Mg^2+^-free buffers, respectively. In order to drive KRAS populations towards the fully open state, a Mg^2+^-free + EDTA-containing buffer was used to scavenge any residual magnesium ions. To drive the equilibrium towards the closed state, a Mg^2+^-containing buffer (10 mM MgCl_2_) was used. As can be seen in Fig. 2C, the affinity of antibody binding increased by sixfold in Mg^2+^—free compared to Mg^2+^-containing buffers. One-way ANOVA with Turkey’s multiple comparison test was performed showing a statistically significant difference (*p* < 0.05) in binding of 4B8 (EC50) between the 1 mM EDTA and the 10 mM MgCl_2_ groups for each of the proteins except G12D. Within the Mg^2+^-free group, there was no statistically significant difference in binding of 4B8 to any of the four proteins. In the 10 mM MgCl_2_ group, there was a statistically significant difference (*p* < 0.05) in binding of 4B8 between WT and G13D, WT and G12D, G13D and G13D 2–169, and G12D and G13D 2–169. In the 10 mM MgCl_2_ buffer, we found that the antibody binds G13D and G12D with higher affinity than G13D 2–169 proposing that the methionine in the first position precludes mAb binding in Mg^2+^ containing media.

### Cellular uptake, distribution, and endosomal escape of the monoclonal antibody in HCT 116 cells

Recent evidence has demonstrated that modified and naked full length IgG antibodies are taken up and distributed in the cytoplasm in KRAS-mutated cancer cells through macropinocytosis/early endosomal escape and direct cell-penetrating mechanisms^29,35–37^. The same studies have also demonstrated that the anti-KRAS activity of these IgG antibodies are maintained in the cytoplasm, suggesting that their structural integrity remains intact. In our study, the cellular uptake of the monoclonal antibody was elucidated using 2 and 4-colour super-resolution microscopy (120 nm resolution) in a G13D-mutated, colorectal cancer (CRC) cell line (HCT 116), known to exhibit macropinocytosis^38^. In summary, free antibody is detected to be present in the plasma membrane, in the cytosol, and shows colocalization to formed plasma membrane vesicles, and to a dextran-based macropinocytosis marker, indicating a plausible macropinocytosis uptake scheme as shown in Supplementary Figs. S3, S4, Supplementary Video S6, Video S7.

### Effects of the monoclonal antibody on intracellular signalling

**Figure 3:**
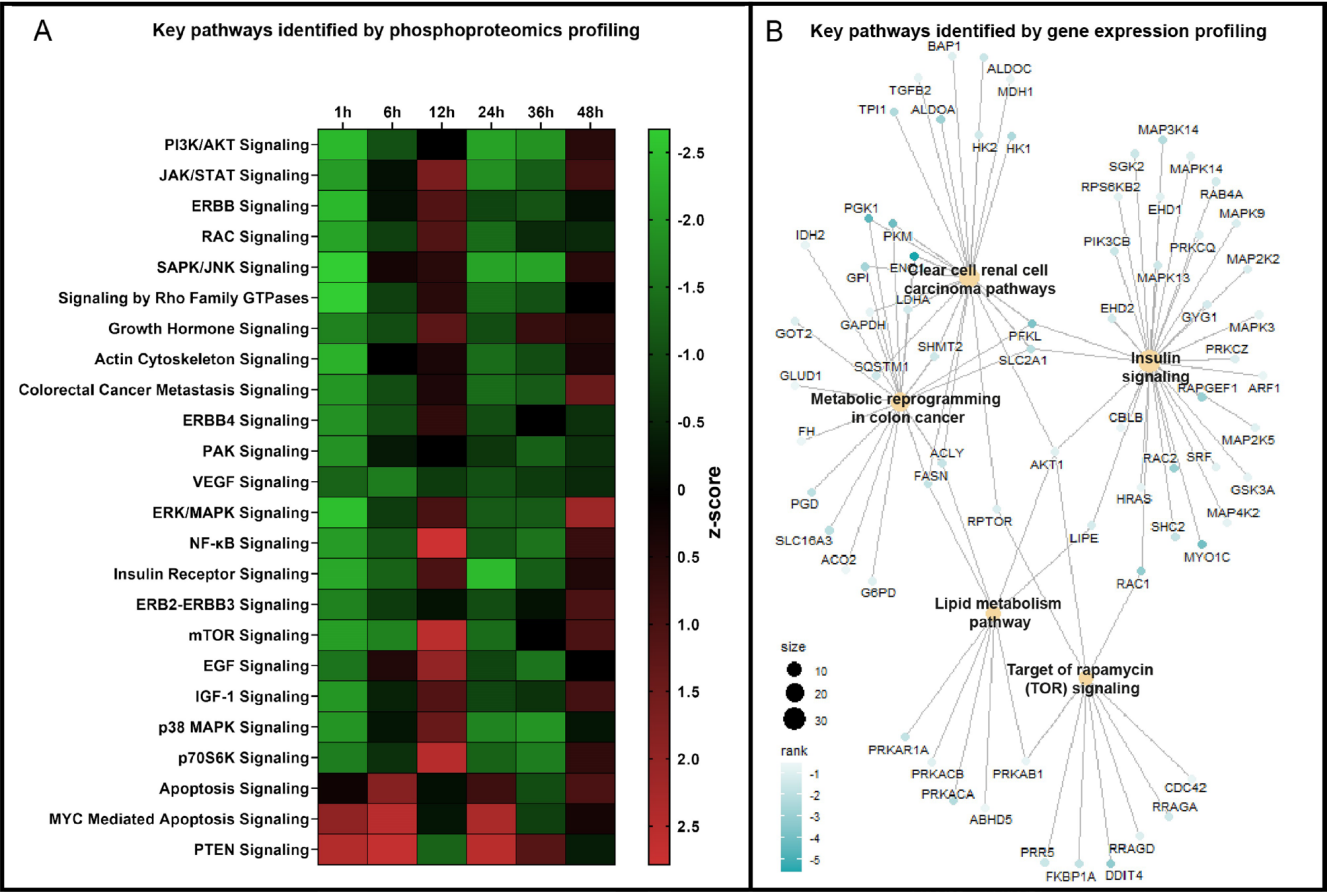
Phosphoproteomic and gene expression profiling of 4B8 showing downregulation of KRAS signalling. (**A**) Heat map of pathways affected by 33 nM 4B8 after treatment of HCT 116 cells for 1 h, 6 h, 12 h, 24 h, 36 h and 48 h, normalized to isotype control. Green colour (negative z-score) indicates inhibition and red colour (positive z-score) indicates activation of the pathway. (**B**) Cnet plot gene expression profiling of HCT 116 cells after 24 h treatment with 33 nM 4B8 showing downregulated KRAS-related pathways affected in 4B8 treated cells, compared to isotype control-treated cells. Only core-enriched genes are displayed, and rank represents the weighted value including log2fold change and *p*-value for each gene.

To investigate whether the antibody could bind and inhibit the fully open KRAS in cells, phosphoproteomic and transcriptomic studies were performed. In the phosphoproteomic study, HCT 116 cells were treated with the antibody or isotype control antibody (33 nM) for 1, 6, 12, 24, 36, and 48 h. After antibody treatment at the indicated time points, cells were lysed, and the lysates were probed against 305 proteins (phosphorylated and non-phosphorylated forms) involved in intracellular signalling. Out of those, 212 proteins were found to have significantly altered phosphorylation levels after antibody treatment for 1 h (*p* < 0.05, data not shown). We observed that the phosphorylation level of several key signalling proteins involved in canonical KRAS/MAPK signalling, including but not limited to, B-Raf, MEK1, PDK1, AKT1, mTOR, MKK4, p38 MAPK, PAK1, MDM2, NFkB^39^ were decreased following 1 h of antibody treatment (Supplementary Fig. S5). At timepoints later than 1 h, the phosphorylation level of most of the proteins appears to change over time with a damped oscillatory behaviour, and at 48 h, little effect was observed (Supplementary Fig. S5). Pathway analysis based on fold change in phosphorylation of proteins was performed using the phosphorylation analysis module in the Ingenuity Pathway Analysis (IPA, from Qiagen). We identified twenty-four signalling pathways as being affected by the antibody over the treatment time course of 1–48 h as shown in Fig. 3A. At the 1 h time mark, pathways positively regulating growth, proliferation, and survival such as ERK/MPAK, PI3K/AKT, mTOR and several receptor tyrosine kinases (e.g., Insulin receptor, IGF1, EGF, ERB2) were significantly affected (*p* < 0.01) and strongly downregulated (from z-score analysis) as shown in Fig. 3A (see also Supplementary Table S3 for individual *p*-values). Like the observation above for single proteins, the pathways in Fig. 3A displayed damped oscillatory behaviour with up-and down-fluctuations in z-scores as a function of time, showing little effect of antibody treatment at 48 h. To display the oscillatory behavior and the anti-correlation between downregulated and upregulated pathways in Fig. 3A as a function of time, a lineplot of the mean Z-score ± SEM of the three upregulated and twenty-one downregulated phosphoproteomic pathways respectively, are shown in Supplementary Fig. S6. Most oncogenic pathways that were downregulated at the 1-h timepoint were also found to be downregulated at 6, 24, and 36 h, but not at 12 and 48 h. Interestingly, a reciprocal dynamical behaviour was observed with the tumour suppressor PTEN, as well as the apoptosis-mediated pathway and the MYC-mediated apoptosis pathway. Apoptosis signalling, MYC-mediated apoptosis signalling and PTEN signalling can all be classified as tumour suppressor pathways since they prevent tumour formation. The results align with previous data where we showed that a polyclonal Ab, based on the same antigen as 4B8 has apoptotic activity in HCT 116 cells^29^.

The PTEN pathway was found to be upregulated by antibody treatment at the 1 h, 6 h, 24 h and 36 h with a dip at 12 h, and 48 h (Fig. 3A, see also Supplementary Table S3 for individual *p*-values at 1 h). The apoptosis-mediated, and MYC-mediated apoptosis pathways responded to a slightly lesser degree compared to the PTEN pathway after antibody treatment (down-regulated at 36 h)–but displayed overall similar time-dependent z-score dynamics (Fig. 3A, Supplementary Fig. S6). Much work remains to understand the oscillatory/multiphasic behavior on pathways caused by 4B8 treatment. However, the work by Relogio et al., comes across as particularly relevant and intriguing for future inspiration and direction^40^. The team used a systems biology approach, integrating experimental, and modelling data from colorectal and other cancer cell lines (including the HCT 116 cell line used herein) and found that the KRAS/MAPK pathway is an important regulator of the circadian system where overexpression of RAS leads to an increase of the circadian period and RAS inhibition results in a shortening of the period. Interestingly, the HCT 116 cell line is intrinsically a strong oscillator, displaying a dampened cosine oscillation with a period of 24.4 ± 0.4 h over 140 h when comparing seven clock genes to Bmal1. The authors propose that the RAS/MAPK signalling pathway modulates the circadian period by influencing the transcriptional activity of the core clock genes CLOCK and Bmal1. Interestingly, looking at phophorylation of key KRAS/MAPK proteins over 48 h (Fig. 3, Supplementary Fig. S6), we similarly observed a dampened wave with a period of about 24 h. Further understanding of this exciting behavior also taking into consideration the doubling time of HCT 116 cells which is about 17 h^41^, as well as dynamics of apoptosis and macropinocytosis will require additional studies.

From the observations above, it appears that several oncogenic pathways related to KRAS/MAPK signalling are down-regulated and tumour-suppressive pathways are upregulated on the level of the phosphoproteome, especially 1 h post mAb treatment. To extend the above observation on intracellular signalling pathways, the effect of the monoclonal antibody on transcriptional programs downstream of KRAS was assessed using transcriptomic profiling. Gene expression profiling of HCT116 cells treated with 33 nM antibody or 33 nM isotype control antibody for 24 h was performed using RNAseq. Gene Set Enrichment Analysis (GSEA) on pathways included in the Wikipathways database, revealed a total of 16 pathways with adjusted *p*-values of < 0.05 as downregulated by antibody treatment (Supplementary Table S4). Five out of 16 pathways that are downregulated by antibody treatment (based on q-values) are KRAS-related i.e., mTOR signalling, clear cell renal cell carcinoma, metabolic reprogramming in colon cancer, lipid metabolism and insulin signalling. These pathways are represented in the Cnet plot displayed in Fig. 3B showing size (number of core-enriched genes), and rank which is the weighted value including log2fold change and *p*-value of each gene. Central nodes in the antibody downregulated pathways mentioned above are PI3K, AKT, RAPTOR, MEK, ERK, RAC, CDC42 which are known mediators of KRAS oncogenic signalling^42^. Taken together, the data suggest a link between the inhibition of phosphorylation of signalling proteins to the inhibition of downstream gene transcription programs caused by antibody treatment.

## Discussion

The main finding in this study is that G13D exists in a fully open state with the switch-I region being fully retracted leaving the P-loop area exposed and druggable. Whereas we do not know the open times for the G13D switch-I open state in a cellular context, the proteases that were used in the proteomics method to identify the switch-I open state had sufficient time to efficiently bind to the epitope^29^. Based on this observation, a P-loop binding mAb was developed and found to inhibit G13D in the cellular context, supporting the notion that G13D is from time to time, fully open and druggable in vivo as well. In support of our structural biology observations, molecular dynamics simulations^43,44^, nuclear magnetic resonance (NMR) spectroscopy experiments^45^ and a small subset of crystal structures reveal a high degree of freedom for movement in the switch regions, and a number of related switch-I fully open state crystal structures have been discovered historically e.g., WT^28^, A146T^20^, V14I^30^, and the G12D mutation (PDB entry: 7F0W). These fully open forms have the P-loop area fully exposed in contrast to conformers that retains Mg^2+^ in the catalytic site as exemplified by D33E^26^, A59G^26^, and G12D^27^. Additionally, the data provided herein suggests that a monoclonal antibody, in a similar sense as other anti-KRAS mAbs and pAbs^29,35–37^, is taken up by KRAS-mutated cancer cells by macropinocytosis^24,46–51^. Furthermore, Kim et al., described a small molecule pan-KRAS inhibitor binding to GDP-bound KRAS. The authors demonstrated effective inhibition of several RAS mutants, including G13D, and A146V/T, as well as WT KRAS suggesting that KRAS oncoproteins are dependent on nucleotide exchange for activation^24^.

**Figure 4:**
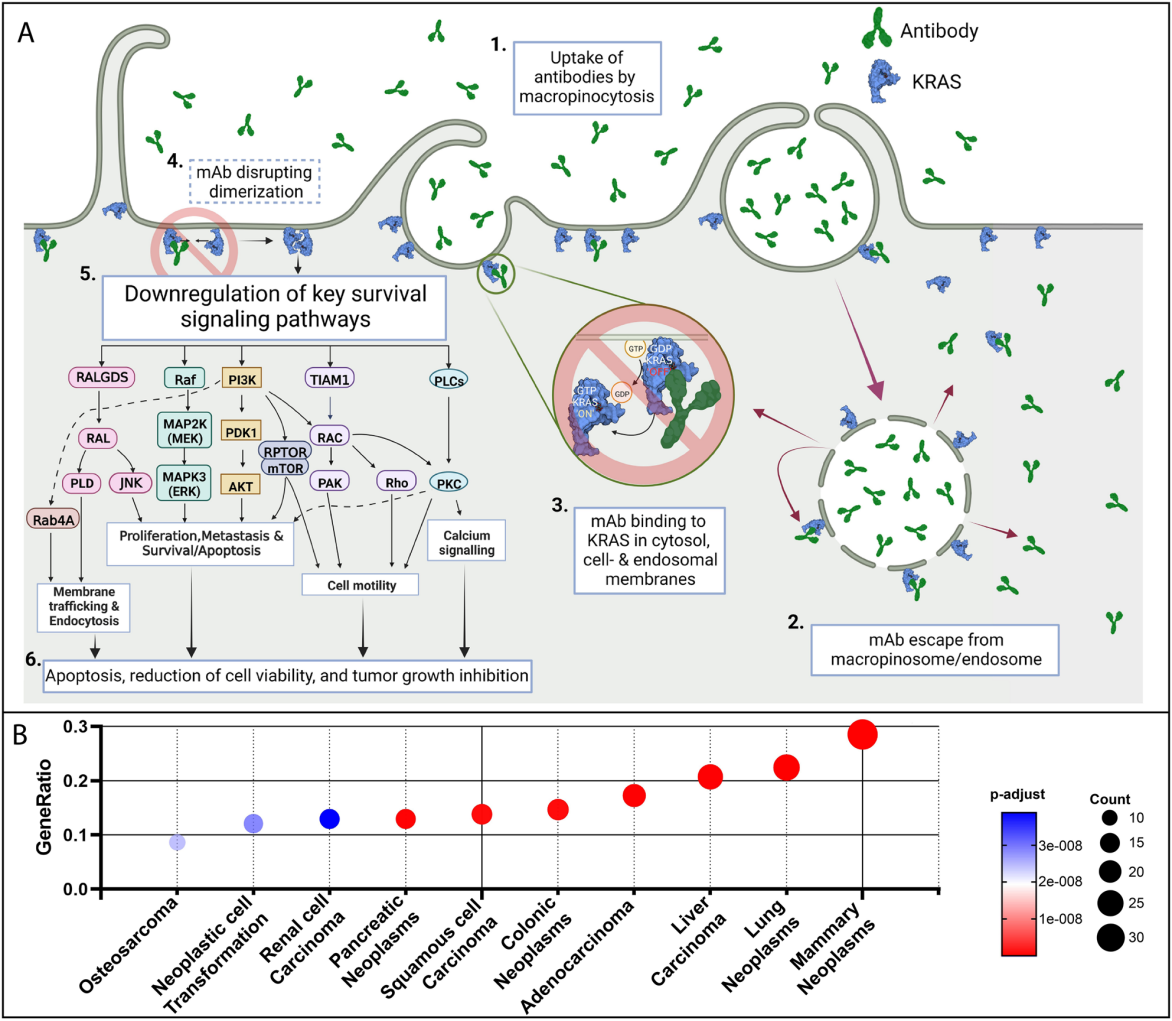
Proposed mechanism of action and plausible disease relevance of 4B8. (**A**) Schematic representation of (1) internalization of antibodies by KRAS-mutated cancer cells through macropinocytosis, followed by (2) escape from macropinosomes/endosomes and (3) interaction with KRAS, (4) possibly through disrupting dimerization, leading to (5) downregulation of key signalling pathways and (6) apoptosis and tumour growth inhibition of G13D-mutated tumours. Figure created with BioRender.com. (**B**) Top 10 diseases associated to phosphorylated proteins displaying biological significance. Gene-disease association database (DisGeNET) was used to identify over-represented diseases based on significantly phosphorylated proteins (ratios of 4B8 treated HCT 116 cells compared to isotype at 1 h after treatment showing up or down regulation with 1.3-fold change were considered as biologically significant).

KRAS is trafficked between the plasma membrane and endosomal compartments at a very high rate^52,53^, and it seems reasonable to hypothesize that local high concentrations of both KRAS and antibody are to be found both at the endosomal membrane and at plasma membrane compartments (see Fig. 4A). The mAb appears to preferentially bind KRAS mutants in a switch-I open, P-loop exposed state. We confirmed that the monoclonal antibody downregulates key oncogenic signalling pathways as demonstrated using both proteomic and transcriptomic pathway analyses in G13D-mutated HCT 116 cells (see Fig. 3). It is plausible that some of these effects are caused by the antibody binding to KRAS in the switch-I out conformation, preventing either effector binding or KRAS dimerization at the plasma membrane level, the latter can lead to e.g., decreased RAF1 activation^14–17^.

Figure 4B display several diseases in the disease association database DisGeNET^54^ as being significantly associated with the pathway signature affected by antibody treatment of cancer cells. Interestingly, these include not only classical KRAS-driven malignancies such as lung, pancreatic and colorectal cancer, but also mammary neoplasms which are not known to harbour KRAS mutations. However, a subset of triple negative breast cancer (TNBC) patient samples has enriched KRAS signalling gene sets^55^. In 2020, colorectal cancer (CRC) tallied almost 2 million new cases and 1 million deaths, representing the third highest mortality rate of all cancers worldwide^56^. About 50% of all CRC is driven by missense mutations in KRAS. 90% of these KRAS mutations are found at codon 12 and 13 and there are currently no drugs available targeting these mutations. Codon 13 mutations in CRC amounts to nearly 20%, thus representing a major count among these patients^57^ and has been associated with lower survival^21–23^. Likewise, codon 146 mutations seem to be associated with aggressive disease^58^. The present study adds to the mounting evidence that anticancer therapies potentially can be devised using naked and modified IgG’s as tools to shut down oncogenic KRAS signalling^29,37,59^. Given the importance of bringing new medicines to patients suffering from KRAS-driven malignancies not the least in CRC, the results described herein warrant further investigation.

## Methods

### Generation of monoclonal antibodies

BALB/c and C57/BL6 mice were immunised with G13D peptide (CGAGDVGKSALTI) linked to keyhole limpet hemocyanin (KLH). Immune responses were evaluated using ELISA against the cognate peptide. Spleen cells from mice with positive immune response were fused with myeloma cells to produce hybridoma cells. Hybridomas were screened by ELISA to obtain positive clones. Positive hybridomas were then used to produce monoclonal antibodies and the antibodies purified by standard procedure using protein A column. cDNA from the hybridomas was used to amplify and sequence variable light (VL) and variable heavy (VH) regions of individual clones. Unique mAbs were identified and used for production of recombinant antibodies using standard methodologies. More specifically, recombinant mAb was produced in a CHO cell line.

Antibody generation was performed by Innovagen AB, Lund, Sweden.

### Measurement of binding affinity by ELISA

Experiments comparing different KRAS peptide variants; Maxisorp Nunc-Immuno Plates (Thermo Scientific) were coated with WT (CGAGGVGKSALTI) or G13D (CGAGDVGKSALTI) or G12D (CGADGVGKSALTI) peptide (1 µg/ml) in PBS containing 155 mM NaCl, 3 mM Na2HPO4, and 1 mM KH2PO4 and incubated overnight at 4 °C.

Plates were washed with PBS containing 0.05% Tween and incubated 1 h at RT in the presence of blocking buffer (3% (w/v) BSA in PBS). 4B8 in TBS + 1% BSA was added at the indicated concentrations and incubated for 1 h at RT. Plates were washed with PBS containing 0.05% Tween. Then, anti-mouse IgG secondary antibody conjugated to HRP (in PBS + 1% BSA and 0.05% Tween), was added, and incubated for 1 h at RT. Plates were washed with PBS containing 0.05% Tween followed by addition of 100 µl per well of TMB and incubated for 10 min. The reaction was stopped with 1 M HCl, and the absorbance was measured at 450 nm.

Experiments comparing KRAS protein ± Mg^2+^; WT, KRAS G12D, KRAS G13D (1–169) or a N-terminally truncated version (2–169) of KRAS G13D protein (see below) were coated onto ELISA plates at 20 ug/ml, overnight at 4 °C. Plates were washed and then blocked with 3% (w/v) BSA in PBS, for 1 h at RT. 4B8 was added in either 1% (w/v) BSA, 20 mM Tris, 50 mM NaCl, 1 mM EDTA for 30 min in RT or 1% (w/v) BSA, 20 mM Tris, 50 mM NaCl, 10 mM MgCl2 for 2 h in RT. Secondary antibody (anti-human IgG Antibody, HRP conjugate) was added in 1% (w/v) BSA in PBS-Tween for 1 h in RT. 100 µl per well of TMB was added and incubated for 10 min. The reaction was stopped with 1 M HCl, and absorbance was measured at 450 nm.

### Cloning, expression, and purification of recombinant KRAS4a proteins

Recombinant KRAS (WT, G13D, G13D 2–169 and G12D) was produced by the Protein Science Facility (PSF) at Karolinska Institutet. The coding sequence for human KRAS4a (residues 1–169), with and without the mutations G13D or G12D, was cloned into expression plasmids pNIC28-Bsa4 by standard cloning methods. The generated constructs carried an N-terminal 6xHis tag followed by a tobacco etch virus (TEV) protease cleavage site (ENLYFQ/S). Additionally, for KRAS4a (G13D), part of this construct was subsequently subject to site-specific mutagenesis to alter the sequence at the TEV site to ENLYFQ/GG, followed by KRAS(G13D) residues 2–169. This construct allowed for the generation of N-terminally Gly-Gly Supplementary KRAS4a (G13D) residues 2–169 after TEV cleavage.

Expression and purification of KRAS4a constructs were performed as follows. The final plasmids were transformed into *E. coli* BL21 (DE3)-T1R competent cells harbouring the rare-tRNA expressing plasmid pRARE2. Terrific Broth growth media (TB, modified, Sigma-Aldrich) supplemented with kanamycin (100 µg/ml) and chloramphenicol (34 μg/ml), and glucose (0.4%) was inoculated with fresh transformants and grown in a shaking incubator overnight at 30 °C. The starter culture was then used to inoculate the main culture in TB supplemented with 1% glycerol and kanamycin (50 μg/ml). The cultures were grown at 37 °C until an OD_600_ of 2 was reached, when the temperature was lowered to 18 °C. Protein expression was induced by the addition of IPTG (ThermoFisher Scientific, Waltham, MA, USA) to a final concentration of 0.5 mM and expression was continued for 16–20 h. Cells were harvested by centrifugation (10 min at 4500×*g*, 4 °C), re-suspended in IMAC lysis buffer (100 mM HEPES, 500 mM NaCl, 10% glycerol, 10 mM imidazole, 0.5 mM TCEP, pH 8.0) supplemented with nuclease (in-house production) and complete EDTA-free protease inhibitor cocktail (Roche, Basel, Switzerland), and disrupted by sonication (4 s/4 s 4 min, 80% amplitude, Sonics Vibracell-VCX750, Sonics & Materials Inc., Newtown, CT, USA). Lysates were centrifuged at 49,000×*g* for 20 min at 4 °C. The supernatants were filtered (Nalgene™ Rapid-Flow™ Bottle top filters, 0.45 µm, ThermoFisher Scientific, Waltham, MA, USA) before loading onto an IMAC HisTrap HP 5 ml column (Cytiva, Little Chalfont, UK), mounted on an ÄKTA Xpress FPLC system (Cytiva, Little Chalfont, UK). After washing with wash buffer 1 (20 mM HEPES, 500 mM NaCl, 10% glycerol, 10 mM imidazole, 0.5 mM TCEP, pH 7.5) and wash buffer 2 (20 mM HEPES, 1 M NaCl, 10% glycerol, 50 mM imidazole, 0.5 mM TCEP, pH 7.5), the bound protein was eluted with elution buffer (20 mM HEPES, 500 mM NaCl, 10% glycerol, 500 mM imidazole, 0.5 mM TCEP, pH 7.5), and further purified by size exclusion chromatography using a HiLoad 16/60 Superdex 75 preparative grade column (Cytiva, Little Chalfont, UK) pre-equilibrated with gel filtration buffer (20 mM HEPES, 300 mM NaCl, 10% glycerol, 2 mM MgCl_2_, 2 mM TCEP, pH 7.5).

Selected fractions were analysed by SDS-PAGE. Fractions containing KRAS4a protein were pooled, and His-tagged TEV protease (in-house production) was added in a 1:30 molar ratio. After 16 h incubation at 8 °C, the cleaved His-tag and TEV protease were removed by reverse IMAC using a 2 ml HisTrap column (Cytiva, Little Chalfont, UK). The flow-through containing the cleaved KRAS4a protein was collected, the protein was concentrated using a Vivaspin concentration filter unit (10 kDa cut off, Sartorius, Göttingen, Germany) and the buffer was exchanged back to gel filtration buffer using a PD-10 column (Cytiva, Little Chalfont, UK). The final concentration 11 mg/ml was calculated from 280 nm absorbance (NanoDrop™, ND-1000 Spectrophotometer, Thermo Scientific, Waltham, MA, USA) using a theoretical extinction coefficient. Finally, the protein was flash frozen in liquid nitrogen and stored at − 80 °C.

### Protein crystallization and crystal harvesting

We crystallised 11 mg/ml KRAS-G13D in 20 mM HEPES pH 7.5, 300 mM NaCl, 2 mM MgCl2, 10% v/v glycerol and 2 mM TCEP, using a mosquito robot, 96-well MRC plates, and two different drop ratios (protein + well) and crystallization buffers either 2.4 M sodium malonate pH 7 mixed 150 + 150 nl (8BLR) or 1.4 M sodium–potassium phosphate pH around 8.7 mixed 100 + 200 nl (8CPR). First sodium–potassium phosphate crystals appeared overnight while malonate crystals appeared after 11 days in 20 °C and both conditions were harvested at day 20. The rod-shaped sodium–potassium phosphate crystals were significantly smaller 20 × 20 × 50 µm as compared to the sodium malonate crystals being 110 × 110 × 130 µm. Both phosphate and malonate crystals were cryo-protected using a cryo solution consisting of well components and an additional 20% v/v Glycerol and 300 mM NaCl. 500 nl cryo-solution was added to the crystal droplet right before the crystals were picked up using dual-thickness micro loops and flash-frozen in liquid nitrogen.

### X-ray data collection and 3D structure refinement

X-ray diffraction datasets were collected at MAX IV BioMAX beamline^60^ in Lund Sweden to 1.4 Å resolution for the malonate crystals and 2.0 Å for the sodium–potassium phosphate crystals. The KRAS4a-G13D mutant crystallised in space group P321 (150) with cell parameters 78.1 78.1 56.2 90 90 120 in malonate, 76.4 76.4 55.9 90 90 120 in phosphate, and one molecule in the asymmetric unit. At BioMAX diffraction data collection we rotated the crystal 0.1 degrees per image and collected 3600 images at 30% transmission to achieve 360 degrees of diffraction data processed by XDSAPP3^61,62^. The sodium-malonate crystals diffracted to 1.4 Å in all directions (isotropically) while the sodium–potassium phosphate crystals diffracted anisotropically to 2.0 Å in the best direction and around 2.4 Å in the worst directions and was therefore elliptically truncated using staraniso web server from Global Phasing (staraniso.globalphasing.org). Elliptical truncations filter out the best available data in the 2.0–2.4 Å region but completeness get significantly lower than for spherically truncated datasets—see Supplementary Table S1. The 3D structure of the KRAS4a-G13D mutant was determined by molecular replacement using the previously observed fully open form of KRAS4a (6M9W) as search model. For scaling, molecular replacement and refinement, software from the CCP4 suite^63^ was used such as Aimless^64^, Phaser^65^, Refmac5^66^ and Coot^67^. The malonate structure was refined to convergence with anisotropic b-factors and final R/Rfree = 11.7/14.4 and deposited in the protein data bank with accession number 8BLR. The sodium–potassium phosphate structure was refined with isotropic b-factors to final R/Rfree = 0.179/0.248 and deposited in the protein data bank with accession number 8CPR.

### Molecular docking

Antibody structure prediction functionality^68^ of Maestro BioLuminate, Schrödinger Suites of Programs v 2021-3 was used to design a homology model of 4B8 with the amino acid sequences for the light and heavy variable regions as the input. Separate templates were chosen for the light and heavy chains with PDB IDs 4HZA, chain L and 4KUZ, chain H^69^, correspondingly. The sequence coordinates of the CDR regions were modified if the automatically assigned coordinates deviated from the ones identified experimentally. Five different models of the CDR regions were generated. Both the Fv and the full antibody (two symmetric Fab and Fc regions) models were generated, using the structure of human IgG1, PDB ID 1HZH^70^. Prime de novo loop prediction functionality^68^ was used to refine the structures of the CDR regions, as those showed the sequence similarity less than 0.7.

The five Fv models for 4B8 were subsequently docked to KRAS crystal structures with PDB IDs: 8BLR (G13D mutant of KRAS4a (2–169) bound to GDP with the switch-I in fully open conformation) and 6M9W (Mg-free KRAS4a bound to GDP with the switch-I in fully open conformation) and 7F0W (KRAS-G12D bound to GDP with switch-I fully open conformation). A set of moderate attractive restraints, with a 50% increase of the scaling factor for the attractive potential, was introduced that included residues 10–21 of KRAS used for the epitope derivation. Docking was performed with Piper^71^, BioLuminate, Schrödinger Suites of Programs v 2021-3. Each docking simulation performed 70,000 rotations and returned 30 best binding poses, which were subsequently analysed for protein–protein interactions with USCF Chimera program^72^.

### Cell culture

HCT 116 (G13D, colon carcinoma) was purchased from The European Collection of Authenticated Cell Cultures General Collection (ECACC) (No. 91091005) and were cultured in Mc Coy’s medium (Gibco, Invitrogen) supplemented with 10% Foetal Bovine Serum (FBS) (Gibco, Invitrogen), 1% penicillin/streptomycin (PeSt) (Gibco, Invitrogen) and 1% l-glutamine (Gibco, Invitogen).

### Elucidation of antibody uptake using super-resolution microscopy

To elucidate the intracellular uptake of 4B8 antibody into HCT 116 cells, 4-colour fluorescent labelling experiments were performed. Briefly, 4B8 were labelled with Alexa Fluor 488 using an Alexa Fluor 488 protein labelling kit (catalog no. A10235, Thermo Fisher Scientific), the cells were treated with 10 µg/ml 4B8 and 75 µg/ml pHrodo™ Red Dextran (Invitrogen™, cat. No. P10361), after 24 h cells were washed and counterstained with Hoechst 33342 (Invitrogen™, cat. No. H21486) and CellMask™ Deep Red Plasma Membrane Stain (Invitrogen™, cat. No. C10046) and immediately imaged using a Zeiss LSM 980 confocal microscope, equipped with a 63× objective 1.4NA that gives a typical resolution of 180 nm laterally. By employing the Airy scan 2 detector, a resolution of approximately 120 nm was achieved through deconvolution. A typical voxel/sampling rate of 71 nm was used for large region imaging, to give the best balance of whole cell information and viewing area. A voxel/sampling 57 nm was employed for zoomed regions, to stay above the conventional Nyquist sampling of 2.1x.

### Phosphoproteomic analysis of intracellular signalling after treatment of HCT116 cells with monoclonal antibody

Phosphorylation degree of a diverse set of proteins involved in intracellular signaling was determined using an array of antibodies against total and phosphorylated forms of these proteins (cell signaling phospho-antibody array, catalogue # PCS300, Full Moon Biosystems, Sunnyvale, CA, USA). HCT 116 cells were treated with 33 nM (5 µg/ml) isotype control or 4B8 for various timepoints, proteins were extracted and labelled with biotin, each sample was applied to an antibody array and the bound protein was detected with Cy3 labelled streptavidin. Ratios of phosphorylated protein (normalized to total protein) from 4B8 treated cells to isotype control treated cells were used to calculate fold change in phosphorylation of individual proteins. This was then used to identify pathways affected by 4B8 treatment using Ingenuity Pathway Analysis (IPA) software (Qiagen) and using the Bioconductor R package clusterProfiler (v3.16) on R (v 4.2.1).

### Assessment of differential gene expression caused by 4B8 in HCT116 cells

HCT 116 cells were treated with 5 µg/ml isotype control or 4B8 for 24 h in triplicates. RNA was extracted using total RNA purification kit (catalogue# 17200, Norgen Biotek) and cDNA library was prepared using QuantiSeq 3ʹ mRNA-Seq Library Prep Kit FWD (Catalogue# 015.24, Lexogen). A Universal Human reference sample (Catalogue# QS0639, ThermoFisher Scientific) was included in the library preparation as a positive control sample, Rnase free water was included as no template control. The cDNA libraries were sequenced using NestSeq500 (Illumina) using the NextSeq 500/550 high output kit v2.5 (Catalogue# 20024906, Illumina) with a 1 × 75 bp read length. The depth of sequencing was 6–8 million reads per sample.

Differential gene expression (DGE) analysis was done in DESeq2 v.1.34.0 applying pre-filtering of genes with < 10 counts, ‘apeglm’ for shrinkage estimator and variance stabilizing transformations (VST) for data transformation. To test for differential expression, raw counts were operated on. Counts per gene were normalised across samples within each comparison. Gene Set Enrichment Analysis (GSEA) was run in R version 4/1/2 using Bioconductor packages clusterProfiler v.4.2.1 and ReactomePA v.1.38.0. Gene sets included in the analyses are based on pathways included in the database WikiPathways. Experiments were performed by TATAA Biocenter, Gothenburg, Sweden.

### Supplementary Information

Supplementary Information.

Supplementary Video 1.

Supplementary Video 2.

Supplementary Video 3.

Supplementary Video 4.

Supplementary Video 5.

Supplementary Video 6.

Supplementary Video 7.

## Data Availability

All data needed to evaluate the article are present in the paper and/or the Supplementary Materials. Antibodies can be produced upon request under a material transfer agreement.
