# Supplementary material for: Novel druggable space in human KRAS G13D discovered using structural bioinformatics and a P-loop targeting monoclonal antibody
Source: Sci Rep. 2024 Aug 23;14:19656. doi: 10.1038/s41598-024-70217-9 (PMC11344056; doi:10.1038/s41598-024-70217-9)
Supplement: Supplementary file 1 — Supplementary Information. [file 41598_2024_70217_MOESM1_ESM.docx]

Supplementary Information

Supplementary Statistics - Chi-test for the docking simulations

Supplementary Methods - Rationale for elucidation of antibody uptake using super-resolution microscopy: Labelling and identification strategy.

Supplementary Results - Cellular uptake, distribution, and endosomal escape of the monoclonal antibody in HCT116 cells

Supplementary Figures S1-S6

Table S1-S4

Video S1-S6

**Supplementary Statistics:**

**First Chi-square test for the docking simulations of antibody to different KRAS structures**

For the antibody, 4B8, 5 models with different conformations of CDR regions were created. Docking simulations were performed for three different KRAS structures with the following PDB IDs: 8BLR, 6M9W, 7F0W; for each structure docking was performed with and without a cofactor, GDP. Each docking run returned 30 best docked poses. Those poses have been analyzed in terms of the percentage interacting with the region of interest (epitope) on KRAS. The docking simulations yielded the following results:

**--**

**For 8BLR (G13D):**

- With GDP: 44% binding (66 out of 150 poses)
- Without GDP: 69% binding (103.5 out of 150 poses)

Observed Frequencies:

|  | **Interacting** | **Not Interacting** | **Total** |
| --- | --- | --- | --- |
| With GDP | 66 | 84 | 150 |
| Without GDP | 103.5 | 46.5 | 150 |
| Total | 169.5 | 130.5 | 300 |

### **For 6M9W (wt):**

- With GDP: 14% binding (21 out of 150 poses)
- Without GDP: 42% binding (63 out of 150 poses)

#### Observed Frequencies:

|  | **Interacting** | **Not Interacting** | **Total** |
| --- | --- | --- | --- |
| With GDP | 21 | 129 | 150 |
| Without GDP | 63 | 87 | 150 |
| Total | 84 | 216 | 300 |

### **For 7F0W (G12D):**

- With GDP: 17% binding (26 out of 150 poses)
- Without GDP: 25% binding (38 out of 150 poses)

#### Observed Frequencies:

|  | **Interacting** | **Not Interacting** | **Total** |
| --- | --- | --- | --- |
| With GDP | 26 | 124 | 150 |
| Without GDP | 38 | 112 | 150 |
| Total | 64 | 236 | 300 |

Expected frequencies for antibody binding in all three PDB structures' cases was calculated based on the total interactions observed for each condition, considering the total number of docking poses. If we assume that binding is not affected by the presence of the co-factor then we get:

#### Chi-Square Calculation:

χ^2^ (8BLR) =(66−75)^2^/75+(103.5−75)^2^/75 = 11.9

χ^2^ (6M9W) = (21−75)^2^/75+(63−75)^2^/75 = 40.8

χ^2^ (7F0W) = (26−75)^2^/75+(38−75)^2^/75 = 51.4

Based on the calculated χ^2^ values, given that the degree of freedom is equal to 1, we here show that there is a significant difference in antibody binding to KRAS structures depending on the presence or absence of the co-factor, for all three KRAS structures tested.

----

**Second Chi-analysis:**

To determine if there is a statistically significant difference in antibody binding to the three different KRAS structures (8BLR, 6M9W, 7F0W) regardless of the presence of GDP, we can combine the percentages of binding for each structure and compare these combined binding rates.

### Combined Binding Percentages

We first calculate the combined binding percentages for each KRAS structure by averaging the percentages with and without GDP.

- **8BLR**: (44%+69%)/2=56.5%
- **6M9W**: (14%+42%)/2=28%
- **7F0W**: (17%+25%)/2=21%

These combined percentages represent the overall binding rates of the antibody to each KRAS structure.

### **Statistical Analysis: Chi-Square Test**

We performed a chi-square test to determine if there are significant differences in the binding frequencies to the three KRAS structures.

1. **Observed Frequencies**

Calculate the number of interactions based on the combined percentages out of 300 docking poses (150 for with GDP and 150 for without GDP).

- - **8BLR**: 56.5%×300=169.5 interactions
  - **6M9W**: 28%×300=84 interactions
  - **7F0W**: 21%×300=63 interactions

Summarize the observed frequencies:

| **Structure** | **Interacting** | **Not Interacting** | **Total** |
| --- | --- | --- | --- |
| 8BLR | 169.5 | 130.5 | 300 |
| 6M9W | 84 | 216 | 300 |
| 7F0W | 63 | 237 | 300 |
| Total | 316.5 | 583.5 | 900 |

1. **Expected Frequencies**

Calculate the expected frequencies assuming no difference in binding rates:

- - **Interacting**: (316.5/900)×300=105.5
  - **Not Interacting**: (583.5/900)×300=194.5

For each structure, the expected frequencies are:

| **Structure** | **Interacting** | **Not Interacting** | **Total** |
| --- | --- | --- | --- |
| 8BLR | 105.5 | 194.5 | 300 |
| 6M9W | 105.5 | 194.5 | 300 |
| 7F0W | 105.5 | 194.5 | 300 |
| Total | 316.5 | 583.5 | 900 |

**Chi-Square Calculation**

χ^2^=∑(O−E)^2^/E

For 8BLR: χ^2^=(169.5−105.5)^2^/105.5+(130.5−194.5)^2^/194.5= 59.89

For 6M9W: χ^2^=(84−105.5)^2^/105.5+(216−194.5)^2^/194.5= 6.76

For 7F0W: χ^2^=(63−105.5)^2^/105.5+(237−194.5)^2^/194.5= 26.41

Total chi-square value:

χ2=59.89+6.76+26.41=93.06

Degrees of freedom (df) = (number of groups - 1) = 3 - 1 = 2.

The critical value for 2 degrees of freedom at α = 0.05 is 5.99.

In conclusion, the calculated chi-square value (93.06) is much greater than the critical value (5.99), indicating that there is a statistically significant difference in the binding of the antibody to the three different KRAS structures.

Supplementary Methods:

**Rationale for elucidation of antibody uptake using super-resolution microscopy:**

**Labelling and identification strategy.**

To investigate the intracellular uptake pathway of the 4B8 antibody, a colocalization strategy was established, the goal of which being to elucidate the internalisation and accumulation location of the 4B8 antibody. Fluorescent labels were used to isolate the various cellular components, in addition to utilising fluorescently labelled 4B8 antibody (Alexa).

To probe the internalisation mechanism of the 4B8 antibody, a pHrodo™ Red Dextran was introduced during the antibody exposure period. Due to its propensity to follow pinocytosis pathways Dextran was employed as a conventional endocytosis marker. Upon pinocytosis, the formed vesicle could be tracked due to the pH sensitivity of the dye labelled attached to the Dextran. As the pinocytosis vesicle is trafficked and fused with early endosomes, the pH lowers and the fluorescence increases.

Sub-cellular organelles presenting a single green colour, (Alexa labelled alone) would indicate recent internalised vesicles. Organelles green and red colocalised would indicate vesicles fused with early endosomes. Cytosolic levels of fluorescent antibody were also probed to investigate any possible release from the early endosomes.

Counter stains were used to identify the subcellular compartments, the Cell membrane was labelled with and CellMask™ Deep Red Plasma Membrane Stain and the Cell nuclei identification was performed using the Hoechst 33342 stain.

**Measurement strategy**

Due to the need to spatially identify the sub-cellular organisation of antibody uptake, a super resolution technique, Airy scan confocal, was chosen. The resolution improvement over conventional confocal microscopy (approx. 1.5x) aids in the spatial isolation, while the signal to noise improvement (approx. 4x over conventional confocal), allowed for greater experimentation duration without significant photobleaching, critical in studying potentially low signal subcellular regions and tracking colocalization over minutes.

Using a Zeiss LSM 980 confocal microscope, equipped with a 63x objective 1.4NA gives a typical resolution of 180nm laterally. Employing the Airy scan 2 detector a resolution of approximately 120nm can be achieved through deconvolution. A typical voxel/sampling rate of 71nm was used for large region imaging, to give the best balance of whole cell information and viewing area. A voxel/sampling 57nm was employed for zoomed regions, to stay above the conventional Nyquist sampling of 2.1x.

Using these multicolour data sets at the very high resolution (approx. 120nm), overlap between the fluorescent targets can identify a degree of colocalization between the various subcellular components. This can be more readily observed in higher zoom confocal scans.

To investigate any potential release of the antibody from the endosomes, a study of the fluorescent signal in the cytosol needed to be performed. Analysis of the cytosol without signals from the vesicles, lysosomes, Golgi apparatus, endoplasmic reticulum, poses a significant imaging challenge. To circumvent any unwanted signal from subcellular organelles, a reference frame was taken as the region occupied by the central region of the cell nucleus, while employing the optical sectioning of the confocal microscope. The volume of the open cytosol in this area is quite low, compared to the total cellular volume, however due to the steric nature of the nuclei, significantly reduced the optical contamination of signal due to subcellular trafficking.

Control samples to establish background signals was performed using the same 4 reagents as in all the experiments apart from using unlabelled instead of labelled 4B8 antibody.

Supplementary Results:

**Cellular uptake, distribution, and endosomal escape of the monoclonal antibody in HCT116 cells**

The cellular uptake of the monoclonal antibody was elucidated using 4-colour super-resolution microscopy (120 nm resolution) in a G13D-mutated, colorectal cancer (CRC) cell line (HCT116), as shown in **Fig. S3A.**

The experiments explored antibody uptake and membrane trafficking in organelle-rich regions as shown in **Fig. S3B-G.** The endosomal release of the antibody into the cytoplasm was investigated in the thin-liquid film perinuclear apical and basal volumes (over and under the nucleus, respectively in z-optical axis dimension) as shown in **Fig. S3H-L and** described in further detail in Supplementary Methods. 66nM of monoclonal antibody-labelled with Alexa Fluor 488 (green), was mixed with 10 kDa Dextran, labelled with pHrodo™ Red (red) and subjected to HCT116 cells for 24 hours. CellMask^TM^ Deep Red (purple) was used to stain the plasma membrane, and Hoechst 33342 (blue) was used to stain the nuclei, as shown in **Fig. S3A**. We observed morphological changes that are typical for macropinocytosis, including membrane ruffling and extracellular protrusion of tent poles^1^. These extend outward from the cell membrane and collapse back to form plasma membrane vesicles on the extracellular side, followed by internalisation and intracellular trafficking as seen in **Fig. S3B** and **Supplementary Video S6**. In **Fig. S3B**, examples of tent poles are highlighted with red arrows, large plasma membrane vesicles are shown with white arrows and internalised plasma membrane vesicles/early endosomes are shown with green arrows. The average diameter of internalised plasma membrane vesicles was 0.9 µm (*n*=50, range; 0.5 µm to 1.5 µm), well within the range of macropinosomes^2,3^. Uptake of green fluorescent antibody in internalised macropinosomes and trafficking to early endosomes was determined through intracellular colocalization of antibody, plasma membrane, and pH-activated red dextran as shown in **Fig. S3C**. An exemplary line profile over a region of interest (shown in green in **Fig. S3C**) confirmed colocalization of antibody, plasma membrane and dextran (**Fig. S3D**), and the correlation (Pearson's correlation coefficient) of colocalization in the same region, over a period of 135 seconds is shown in **Fig. S3E-G** and **Supplementary Video S7**. A clear correlation between the Dextran and the antibody colocalization can be observed in **Fig. S3G**, together with a strong correlation between both the dextran (**Fig. S3E)** and the antibody (**Fig. S3F)** with the membrane. To investigate endosomal escape of mAbs into the cytosol, confocal micrographs were taken in a region of the cell largely occupied by the nuclei as exemplified in **Fig. S3H**. This region was chosen for two core reasons: (1) this region occupied by the nuclei bulk, hinders endosomes to be trafficked across the region during the observation period. This is crucial as the signal of interest is cytosolic content only and fluorescence signals from endosomes would interfere with the measurement, and accurately removing each endosome would restrict observational volume. (2) this region is typically a thin (when the cell culture is on a planar surface), and generally does not contain other organelles, allowing for 10’s of µm^2^ of a very thin cytosolic region, removing interference and the need to account for many on-axis out of focus components to the signal. The nuclei were then isolated using image analysis **(Fig. S3I**) and used to establish a binary image mask (**Fig. S3J**). This binary mask was then applied to isolate the signal from the free-in-cytoplasm antibody which was found to be distributed across the volume as shown in **Fig. S3K** and fluorescence signal was calculated per square micrometre in antibody-treated and in unstained cells. The fluorescence signal was determined to be 5.3-fold higher in treated cells compared to unstained controls as shown in **Fig. S3L**. To specifically investigate the location of mAb to subcellular membrane compartments, double labelling experiments using Alexa Fluor 488-labelled mAb (green), and CellMask^TM^ Deep Red (purple) were employed at the same concentrations and protocols as explained above four the four-label approach (see **Fig. S4).**

Supplementary Figures:

Figure S1. ELISA measurements of 4B8 against KRAS peptides 10-21

**

**

ELISA measurements of 4B8 against KRAS peptides corresponding to amino acids 10-21 in wt, G13D and G12D KRAS. No binding was observed to irrelevant peptides. Data is presented as mean ±SEM, *n*=3. There was no statistically significant difference between binding of 4B8 to any of the three peptides.

Figure S2. Molecular docking simulations of 4B8 to KRAS G13D with switch-I in fully open conformation, in different orientations.

**
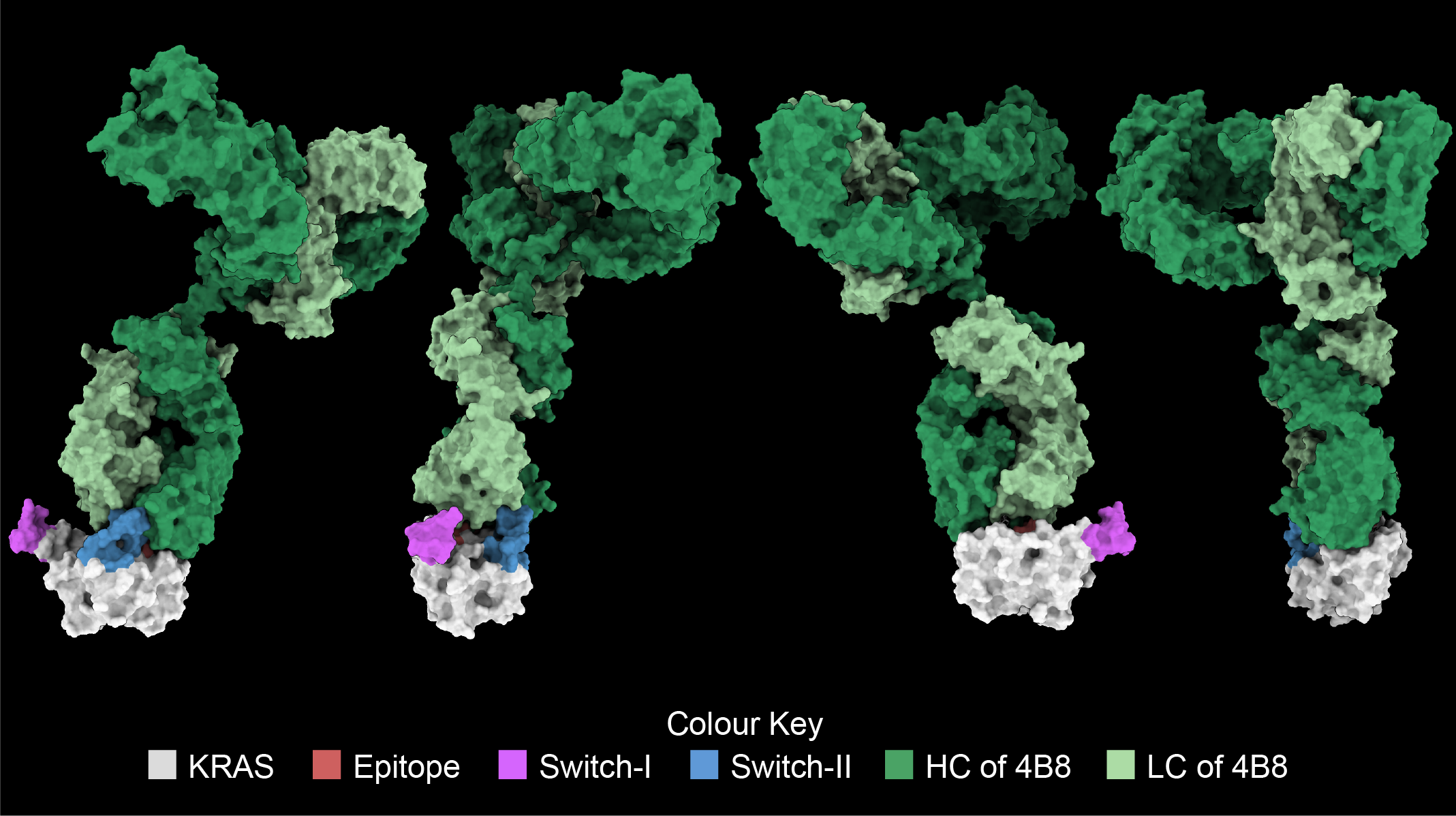
**

Docking pose showing a surface model of antibody 4B8 interacting with the epitope region 10-21 of human KRAS G13D (PDB: 8BLR) protein, in different orientations. Crystal structure of KRAS G13D 8BLR with the switch-I in fully open conformation (grey). Epitope region (aa 10-21, red). switch-I (aa30-40, purple), switch-II (aa 58-72, blue). 4B8 heavy chain (dark green). 4B8 light chain (light green) respectively

Figure S3. Cellular uptake and endosomal escape of 4B8 in HCT116 cells

**
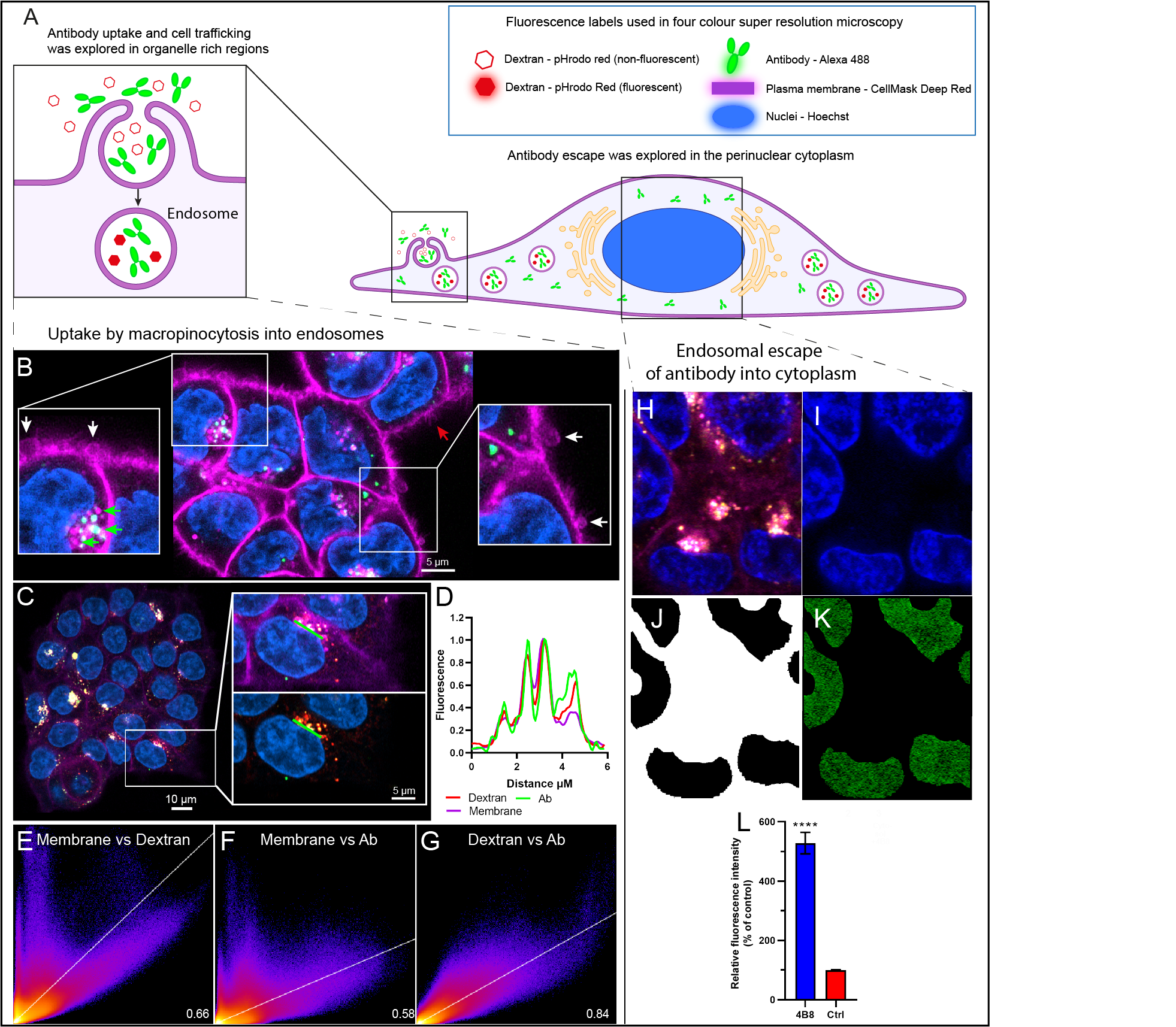

(A)** Schematic drawing displaying measurement strategy to confirm cellular uptake of 4B8 through macropinocytosis, using super-resolution microscopy in HCT116 (G13D) cells, using dextran as an endocytic marker. Antibody (green), plasma membrane (purple), pHrodo™ Red Dextran (red), and nuclei (blue). The left panel schematically shows the strategy used to confirm macropinocytotic uptake through colocalization of antibody with plasma membrane and dextran. The right panel schematically shows the strategy used to confirm endosomal escape by measuring the thin-liquid film perinuclear apical and basal volumes (**B)** Morphological characteristics of macropinosome uptake of 4B8 in HCT116 cells after treatment with 66 nM fluorescently labelled 4B8 (green) for 24 hr., specifically, ‘tent poles’ (red arrow), large plasma membrane vesicles (white arrows), and internalised plasma membrane vesicles/early endosomes (green arrows). (**C)** Intracellular colocalisation of antibody, dextran, and plasma membrane after treatment with 66 nM fluorescently labelled 4B8 and 75 ug/ml of Dextran for 24 hr. Region for line profile is depicted in green. **(D**) Line profile of 4B8 in endosomes; displayed overlap of the antibody (green), plasma membrane (purple) and dextran (red) signals from the line in C. (**E**) Correlation analysis of plasma membrane and dextran signal, (**F**) antibody and membrane signal, and finally (**G**) antibody and dextran signal. (**H)** Example of image region used for evaluation of endosomal escape after treatment with 66 nM fluorescently labelled 4B8 for 24 hr. (**I**) Isolated nuclei from image H. (**J**) Binary mask created from nuclei in I. (**K)** Free antibody signal (green) in cytosol after the binary mask in J was applied to the green channel, to isolate the non-vesicular, fluorescent antibody signal. The image has been enhanced for visualisation purposes. (**L**) Fluorescence signal from 4B8, measured free in the cytosol as defined and exemplified in images H-K, comparing 4B8 treated cells to unstained cells. Statistical significance was determined using a non-parametric (Mann-Whitney) U-test. *n*=342 (4B8), 343 (Ctrl).

**Figure S4. Uptake and distribution of 4B8 in different subcellular compartments in HCT116 cells**


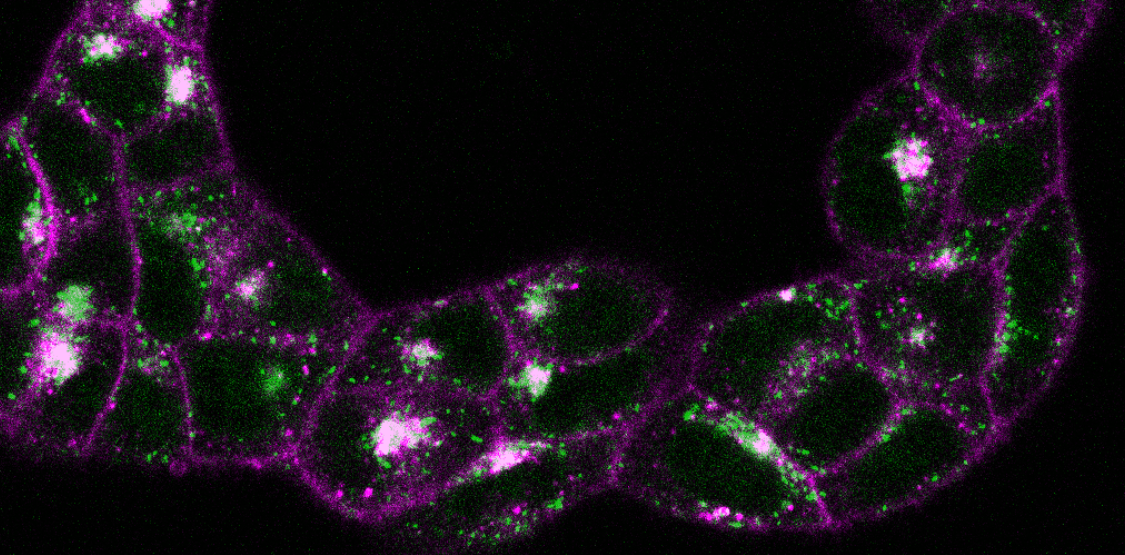


High-resolution confocal image showing uptake and distribution of 4B8 in HCT116 cells after treatment with 66 nM fluorescently labelled 4B8 (Alexa-488, green) for 24 hr. The plasma membrane was stained with Cellmask Deep red (purple).


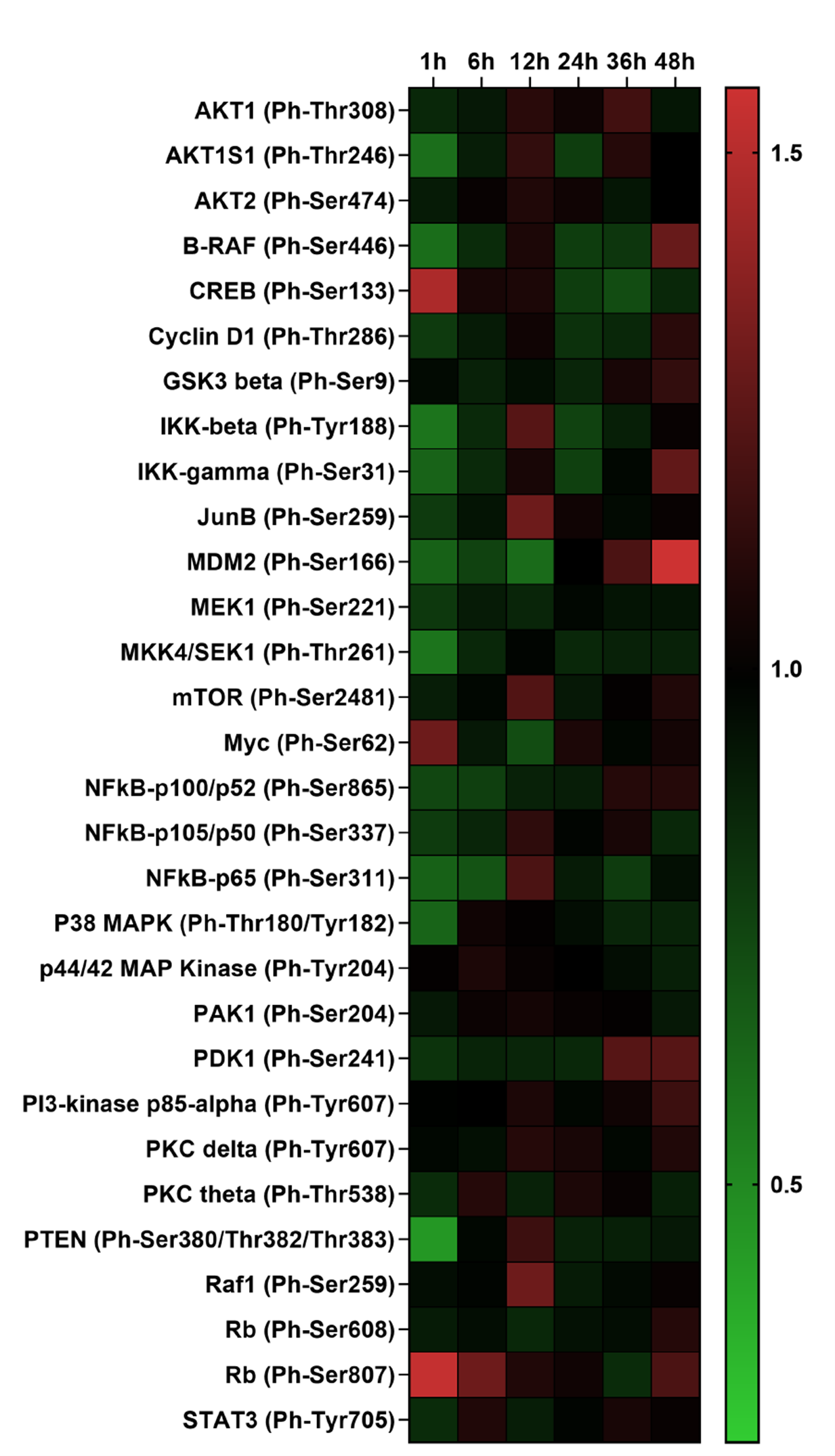
Figure S5. Effects of 4B8 on phosphorylation status of key proteins

The fold-change was calculated from the ratio between phosphorylated and total protein in 4B8- treated HCT116 cells, measured 1-48h after treatment and normalized to isotype-treated HCT116 cells. For further details see materials and methods.

Figure S6. A lineplot displaying the dynamics of upregulated and downregulated phosphoproteomic pathways, measured at 1h, 6h, 12h, 24h, and 48 hours


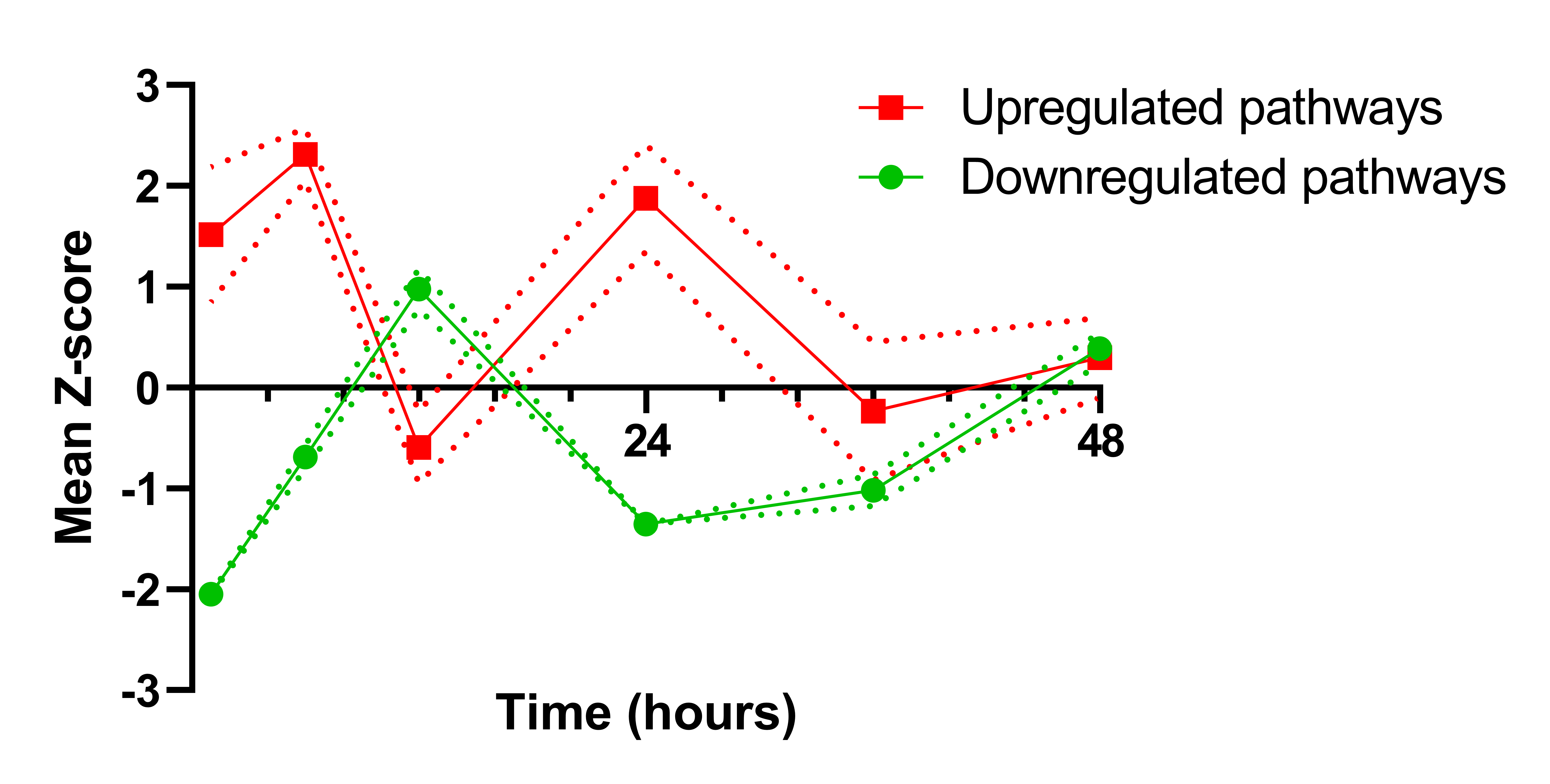


A plot of the mean Z-score ± SEM for upregulated (*n*=3) and downregulated (*n*=21) phosphoproteomics pathways in 4B8- treated HCT116 cells, measured 1-48h after treatment and normalized to isotype-treated HCT116 cells. Upregulated and downregulated are anti-correlated and the amplitudes are highest at early timepoints and goes toward zero at 48 hours.

Table. S1. Data collection and refinement statistics

|  | **KRAS4b-G13D** | **KRAS4b-G13D** |
| --- | --- | --- |
| **Data collection** |  |  |
| PDB ID | 8BLR | 8CPR |
| Space group | P 3 2 1 (150) | P 3 2 1 (150) |
| Cell dimensions |  |  |
| *a*, *b*, *c* (Å) | 78.08, 78.08, 56.20 | 76.41 76.41 55.86 |
| α, β, γ (°) | 90.0, 90.0, 120.0 | 90.0, 90.0, 120.0 |
| Resolution (Å) | 43.2-1.4 (1.42-1.40) | 42.7-2.0 (2.15-2.0) |
| *R*_merge_ | 0.071 (1.632) | 0.172 (1.155) |
| *I* / σ*I* | 20.7 (2.3) | 8.6 (2.1) |
| Completeness spherical (%) | 100.0 (100.0) | 73.3 (19.6) |
| Completeness elliptical (%) | 100.0 (100.0) | 94.8 (88.5) |
| Redundancy | 19.7 (19.9) | 9.7 (9.4) |
|  |  |  |
| **Refinement** |  |  |
| Resolution (Å) | 46.26-1.40 (1.44-1.4) | 42.7-2.0 (2.05-2.0) |
| No. unique reflections | 37 309 (2887) | 9087 (140) |
| *R*_work_ / *R*_free_ | 0.117/0.144 (0.207/0.224) | 0.179/0.248 (0.311/0.394) |
| No. atoms | 1 559 | 1 501 |
| Protein | 1 358 | 1 358 |
| GDP | 28 | 28 |
| Water | 173 | 115 |
| *B*-factors |  |  |
| Protein | 27.9 | 29.6 |
| GDP | 21.6 | 24.3 |
| Water | 46.8 | 41.6 |
| R.m.s. deviations |  |  |
| Bond lengths (Å) | 0.011 | 0.008 |
| Bond angles (°) | 1.627 | 1.453 |

| PDB | Mutant | Nres | N_SSE_ | RMSD | Q-score | Splice variant |
| --- | --- | --- | --- | --- | --- | --- |
| 6BOF:A | A146T | 168 | 13 | 0.1534 | 0.9855 | 2 |
| 6BOF:B | A146T | 168 | 13 | 0.1726 | 0.9848 | 2 |
| 6M9W | WT | 168 | 13 | 0.1426 | 0.9859 | 2 |
| 6MQG | V14I | 167 | 13 | 0.1834 | 0.9903 | 2 |
| 7F0W | G12D | 167 | 13 | 0.3983 | 0.9768 | 2 |
| 7KMR | A59G | 171 | 13 | 0.1821 | 0.9672 | 2 |
| 8BLR | G13D | 168 | 13 | 0.2020 | 0.9836 | 1 |
| 8CPR | G13D | 168 | 12 | 0.5828 | 0.9522 | 1 |
| 8EDY | A146T | 170 | 13 | 0.2057 | 0.9719 | 2 |
| 8EER | A146V | 169 | 13 | 0.1674 | 0.9792 | 2 |

Table S2*.* PDBeFold alignments of fully open form KRAS structures of length 167-171 amino acids. Overall the number of aligned residues was 166, the overall root mean square deviation (RMSD) was 0.4082, overall Q-score being 0.9474, and 12 of 13 Secondary Structure Elements (SSE) aligned.

Table S3. List of key signalling pathways from phosphoproteomic profiling affected by 4B8 treatment of HCT116 cells for 1 hour. Ingenuity Pathway Analysis was performed on phosphoproteins that had at least a fold change of +1.2 or -1.2. Positive z-scores represent activation of the pathway, and negative z-scores represents inhibition of the pathway. *p*-value is based on the overlap of phosphoproteins in our dataset and the pathways in the Ingenuity Pathway Analysis knowledge base.

| **Canonical Pathways** | **z-score** | ***p*-value** |
| --- | --- | --- |
| PI3K/AKT Signaling | -3.13 | 1.23E-32 |
| JAK/STAT Signaling | -2.714 | 1.26E-17 |
| ERBB Signaling | -2.646 | 3.39E-10 |
| RAC Signaling | -2.449 | 1.72E-07 |
| SAPK/JNK Signaling | -2.449 | 0.003795 |
| Signaling by Rho Family GTPases | -2.449 | 1.46E-05 |
| Growth Hormone Signaling | -2.236 | 2.26E-06 |
| Actin Cytoskeleton Signaling | -2 | 0.006065 |
| Colorectal Cancer Metastasis Signaling | -2 | 9.83E-20 |
| ERBB4 Signaling | -2 | 5.07E-05 |
| PAK Signaling | -2 | 0.000414 |
| VEGF Signaling | -1.89 | 1.5E-11 |
| ERK/MAPK Signaling | -1.807 | 2.17E-18 |
| NF-κB Signaling | -1.807 | 1.05E-10 |
| Insulin Receptor Signaling | -1.732 | 9.69E-15 |
| ERB2-ERBB3 Signaling | -1.667 | 3.01E-13 |
| mTOR Signaling | -1.667 | 1.45E-08 |
| EGF Signaling | -1.414 | 5.16E-12 |
| IGF-1 Signaling | -1.414 | 2.57E-11 |
| p38 MAPK Signaling | -1.414 | 2.5E-12 |
| p70S6K Signaling | -1.265 | 8.19E-06 |
| Apoptosis Signaling | 0.535 | 9.95E-22 |
| MYC Mediated Apoptosis Signaling | 2.333 | 2.44E-14 |
| PTEN Signaling | 2.828 | 3.28E-26 |

**Table S4.** List of affected pathways from transcriptomic profiling using Wikipathways after 24h treatment (analysis of a total of 707 pathways). The set size is the total number of genes in the pathway, the enrichment score represents the number of genes which over-represented both at the top and bottom of the gene list, and NES is the enrichment score, normalized to mean enrichment of same-sized random samples. The Q value represents the p-value after multiple hypothesis test correction (between 0 and 1). Core-enrichment are lists of genes that contributed the most to the enrichment set. The leading-edge analysis provides the percentage number of genes contributing to the enrichment score, enrichment signal strength, and where in the list the enrichments score is attained.

| **ID** | **Description** | **Set**  **Size** | **Enrichment**  **Score** | **NES** | **Pvalue** | **p.adjust** | **qvalues** | **core_enrichment** | **leading-edge** |
| --- | --- | --- | --- | --- | --- | --- | --- | --- | --- |
| WP4018 | **Clear cell renal cell carcinoma pathways** | 69 | -0.66 | -2.27 | 8.49E-09 | 5.62E-06 | 5.06E-06 | **MDH1/GAPDH/TGFB2/AKT1/BAP1/RPTOR/LDHA/**  **HK2/SQSTM1/SHMT2/ALDOC/ACLY/FASN/SLC2A1/**  **TPI1/HK1/GPI/ALDOA/PFKL/PKM/PGK1/ENO1** | tags=32%. list=10%. signal=29% |
| WP534 | **Glycolysis and gluconeogenesis** | 31 | -0.78 | -2.31 | 4.89E-08 | 1.42E-05 | 1.28E-05 | **GOT2/LDHA/HK2/SLC2A3/ALDOC/SLC2A1/TPI1/HK1/GPI/ALDOA/PFKL/PKM/PGK1/ENO1** | tags=45%. list=5%. signal=43% |
| WP4290 | **Metabolic reprogramming in colon cancer** | 40 | -0.72 | -2.27 | 6.45E-08 | 1.42E-05 | 1.28E-05 | **GLUD1/ACO2/FH/IDH2/GAPDH/G6PD/GOT2/LDHA/SHMT2/ACLY/FASN/PGD/SLC16A3/SLC2A1/GPI/PFKL/PKM/PGK1/ENO1** | tags=48%. list=6%. signal=45% |
| WP1471 | **Target of rapamycin (TOR) signaling** | 34 | -0.69 | -2.12 | 7.39E-06 | 0.001223 | 0.001101 | **CDC42/PRKAB1/AKT1/RRAGD/RPTOR/RRAGA/PRR5/FKBP1A/RAC1/DDIT4** | tags=29%. list=11%. signal=26% |

| WP4629 | **Aerobic glycolysis** | 11 | -0.89 | -2.02 | 1.06E-05 | 0.001381 | 0.001243 | **LDHA/SLC2A1/TPI1/HK1/GPI/ALDOA/PKM/PGK1/**  **ENO1** | tags=82%. list=3%. signal=80% |
| --- | --- | --- | --- | --- | --- | --- | --- | --- | --- |
| WP5049 | **Glycolysis in senescence** | 11 | -0.88 | -2.02 | 1.25E-05 | 0.001381 | 0.001243 | **GAPDH/G6PD/TP53/LDHA/ALDOC/HK1/PKM/PGK1/ENO1** | tags=82%. list=9%. signal=75% |
| WP1946 | **Cori cycle** | 13 | -0.83 | -1.97 | 5.04E-05 | 0.004762 | 0.004286 | **GAPDH/G6PD/LDHA/SLC2A1/TPI1/HK1/GPI/ALDOA/**  **PGK1** | tags=69%. list=9%. signal=63% |
| WP481 | **Insulin signaling** | 148 | -0.42 | -1.65 | 0.00018 | 0.01489 | 0.013401 | **ARF1/CBLB/HRAS/MAP4K2/GSK3A/MAPK3/SRF/**  **MAP2K5/RPS6KB2/EHD1/AKT1/MAPK14/PRKCZ/**  **MAPK9/MAP2K2/PRKCQ/LIPE/RAB4A/GYG1/EHD2/MAPK13/SGK2/PIK3CB/SHC2/SLC2A1/MAP3K14/**  **RAPGEF1/RAC2/RAC1/PFKL/MYO1C** | tags=21%. list=9%. signal=19% |
| WP3614 | **Photodynamic therapy-induced HIF-1 survival signaling** | 32 | -0.64 | -1.94 | 0.00025 | 0.018422 | 0.016579 | **TP53/LDHA/BNIP3/SLC2A3/EGLN1/SLC2A1/HK1/PFKL/PKM/PGK1** | tags=31%. list=5%. signal=30% |
| WP4949 | **16p11.2 proximal deletion syndrome** | 66 | -0.51 | -1.74 | 0.00033 | 0.021867 | 0.01968 | **NFKB1/TCP1/PPP2R1A/MVP/CDIPT/MAPK3/CCDC6/CCT8/CCT6B/TP53/PPP2CA/PPP2CB/MAZ/PPP2R5D/IGBP1/KIF22/EZR/CCT3/ALDOA** | tags=29%. list=12%. signal=25% |

| WP4566 | **Translation inhibitors in chronically activated PDGFRA cells** | | 42 | -0.57 | -1.81 | 0.000536 | 0.030662 | 0.027596 | **RPS6KA2/PDK1/MAPK3/PIM2/MAP2K5/RPS6KB2/**  **AKT1/MAPK14/MAPK9/RPTOR/MAP2K2/PIK3CB/**  **PRKACA** | tags=31%. list=8%. signal=28% |
| --- | --- | --- | --- | --- | --- | --- | --- | --- | --- | --- |
| WP4540 | **Hippo signaling regulation pathways** | | 68 | -0.50 | -1.72 | 0.000579 | 0.030662 | 0.027596 | **LATS1/GNAI3/TCF7/CTNNB1/GNAQ/PLCB4/CDC42/**  **CDH3/PRKAB1/CDH15/GNAI2/PRKACB/MET/PRKCZ/PRKCQ/FGFR4/PLCB3/TEAD2/PRKAR1A/PRKACA/**  **RHOA/RAC1** | tags=32%. list=16%. signal=27% |
| WP3965 | **Lipid metabolism pathway** | | 25 | -0.66 | -1.87 | 0.00063 | 0.030662 | 0.027596 | **ABHD5/PRKAB1/PRKACB/AKT1/LIPE/ACLY/FASN/**  **PRKAR1A/PRKACA** | tags=36%. list=13%. signal=31% |
| WP2118 | **Arrhythmogenic right ventricular cardiomyopathy** | | 45 | -0.57 | -1.81 | 0.000648 | 0.030662 | 0.027596 | **CACNA1D/ACTB/DSG2/ITGA10/ITGA3/ITGA1/ITGA9/LAMA2/TCF7/ACTG1/CTNNB1/CACNB3/ITGA5/ATP2A2/CACNB4/PKP2/DAG1/DES/ITGB4/ACTN4/JUP** | tags=47%. list=24%. signal=36% |
| WP2436 | **Dopamine metabolism** | 9 | | -0.82 | -1.78 | 0.000966 | 0.042653 | 0.038387 | **MAOB/PRKACB/PPP2CA/PPP2CB/PRKACA** | tags=56%. list=8%. signal=51% |
| WP5116 | **SARS-CoV-2 B.1.1.7 variant antagonises innate immune activation** | 8 | | 0.84 | 1.82 | 0.001134 | 0.046931 | 0.042237 | **IKBKE/TBK1/DDX58** | tags=38%. list=8%. signal=34% |

Supplementary References:

1. Condon, N. D. *et al.* Macropinosome formation by tent pole ruffling in macrophages. *Journal of Cell Biology* **217**, (2018).

2. Jin, J. *et al.* In situ exploration of characteristics of macropinocytosis and size range of internalized substances in cells by 3D-structured illumination microscopy. *Int J Nanomedicine* **13**, (2018).

3. Lim, J. P. & Gleeson, P. A. Macropinocytosis: An endocytic pathway for internalising large gulps. *Immunology and Cell Biology* vol. 89 Preprint at https://doi.org/10.1038/icb.2011.20 (2011).
